# The Cytochrome P450 Monooxygenase Inventory of Grapevine (*Vitis vinifera* L.): Genome-Wide Identification, Evolutionary Characterization and Expression Analysis

**DOI:** 10.3389/fgene.2020.00044

**Published:** 2020-02-18

**Authors:** Songtao Jiu, Yan Xu, Jiyuan Wang, Lei Wang, Xunju Liu, Wanxia Sun, Irfan ali Sabir, Chao Ma, Wenping Xu, Shiping Wang, Muhammad Abdullah, Caixi Zhang

**Affiliations:** Department of Plant Science, School of Agriculture and Biology, Shanghai Jiao Tong University, Shanghai, China

**Keywords:** genome-wide, cytochrome P450 gene superfamily, phylogeny analysis, expression analysis, grapevine

## Abstract

The cytochrome P450 (CYP) monooxygenase superfamily, belonging to heme-thiolate protein products, plays a vital role in metabolizing physiologically valuable compounds in plants. To date, CYP superfamily genes have not yet been characterized in grapevine (*V. vinifera* L.), and their functions remain unclear. In this study, a sum of 236 *VvCYPs*, divided into 46 families and clustered into nine clans, have been identified based on bioinformatics analyses in grapevine genome. The characteristics of both exon–intron organizations and motif structures further supported the close evolutionary relationships of *VvCYP* superfamily as well as the reliability of phylogenetic analysis. The gene number-based hierarchical cluster of CYP subfamilies of different plants demonstrated that the loss of CYP families seems to be limited to single species or single taxa. Promoter analysis elucidated various *cis*-regulatory elements related to phytohormone signaling, plant growth and development, as well as abiotic/biotic stress responses. The tandem duplication mainly contributed to the expansion of the *VvCYP* superfamily, followed by singleton duplication in grapevine. Global RNA-sequencing data of grapevine showed functional divergence of *VvCYPs* as diverse expression patterns of *VvCYPs* in various organs, tissues, and developmental phases, which were confirmed by quantitative real-time reverse transcription PCR (qRT-PCR). Taken together, our results provided valuable inventory for understanding the classification and biological functions of the *VvCYPs* and paved the way for further functional verification of these *VvCYPs* and are helpful to grapevine molecular breeding.

## Introduction

The cytochrome P450 (CYP) monooxygenases constitute a huge and intricate gene superfamily of heme-thiolate proteins, which play important roles in the metabolism of various substrates, containing endogenous compounds, for example, steroids, also xenobiotic compounds including pesticides, drug, and environmental pollutants (Werck-Reichhart and Feyereisen, 2000). The CYP enzyme is probably the most catalytic biocatalyst in nature. According to its functions, CYPs can be roughly classified into two categories in plants. One has a metabolic detoxification function, such as catalyzing environmental toxins, organic dyes, and pesticides to become nontoxic substances (Robert and Douglas, 1991; Werck-Reichhart, 1995). The others participated in the biosynthesis of plant secondary substances, including terpenes, sterols, phytohormones, lignin intermediates, and so on. The CYP genes are widely found in different life forms, including multicellular eukaryotes (animals and plants), unicellular eukaryotes (fungi and protists), and prokaryotes (bacteria and archaea) (Omura, 1999; Ai et al., 2011). In 1969, Frear reported the first plant CYP gene in cotton (*Gossypium hirsutum*). Subsequently, many CYP genes were identified in several plant species, such as *Arabidopsis thaliana* (245) (Paquette et al., 2000), *Medicago truncatula* (151) (Li et al., 2007), *Populus trichocarpa* (310) (Nelson et al., 2008), *Morus notabilis* (174) (Ma et al., 2014), *Ricinus communis* (210) (Kumar et al., 2014), and *Oryza sativa* (326) (Wei and Chen, 2018). To date, more than 20,000 members of the CYP gene family have been characterized and named (Nelson, 2011). However, the amount of CYP genes found in each species is highly varying. Until now, more than 5,100 *CYPs* have been found in plants, and most of them also have been uploaded into a periodically updated Cytochrome P450 Homepage website (http://drnelson.uthsc.edu/CytochromeP450.html). The CYP genes were divided into different clans including six single-family clans (clan 51, clan 74, clan 97, clan 710, clan 711, and clan 727) and four multiple-family clans (clan 71, clan 72, clan 85, and clan 86) according to the criterion of phylogeny and homology (Nelson et al., 1996; Nelson et al., 2004).

In plants, *CYPs* function as a vital regulator in some growth and development processes. For example, cytochrome P450 MAX1 gene, found in *A. thaliana*, *O. Sativa,* and *Solanum lycopersicum*, is necessary for the biosynthesis of strigolactones, mainly involved in regulating the branch of plants (Zhang et al., 2014). The *AtCYP715* is responsible for regulating the maturation of *Arabidopsis* flower, synchronizing volatile emanation, and petal expansion (Liu et al., 2015). CYP703, CYP704, and CYP86 are necessary for the formation of pollen exine and the synthesis of anther cutin (Shi et al., 2015). In *Arabidopsis*, overexpression of *AtCYP78A9* results in seedless and bigger fruits (Ito and Meyerowitz, 2000). In rice, the loss-of-function of *OsCYP78B5* leads to a tremendous embryo (Chen et al., 2015). The CYP members of clan 73, clan 84, clan 98 are responsible for catalyzing the biosynthetic reactions of phenylpropanoid pathway, producing a mass of phenol compound functioning as UV protectant (flavonoids), structural component (suberin and lignin), antimicrobial (isoflavonoids, lignans and coumarins), and antioxidants (polyphenols) (Mizutani and Ohta, 2010). The *CYPs* also participate in stress responses, for instance, salinity, drought, insect pest, oxidative stress, and chemical toxicity. The RNAi lines of *OsABA8ox3* confers drought tolerance in *O. sativa* (Cai et al., 2015). The mutants of CYP96B4/dss1 increase the accumulation levels of abscisic acid (ABA) and ABA-metabolites responding to drought stress (Tamiru et al., 2015). Besides, *OsCYP96B4* may participate in secondary cell wall formation and lipid metabolism in *O. sativa* (Ramamoorthy et al., 2011; Wang et al., 2016). *AtCYP709B1, AtCYP709B2,* and *AtCYP709B3* in *Arabidopsis* are induced under salt stress (Mao et al., 2013). *ZmCYP71C1* and *ZmCYP71C3* are involved in the synthesis of DIMBOA in *Zea mays*, which act as natural defense against bacteria, fungi, and pest (Persans et al., 2001). Until now, only one *VvCYP71BE5* has been functionally characterized (Takase et al., 2015), the majority of *VvCYP* superfamily members have not been investigated in grapevine.

Grapevine (*Vitis* spp.), with processing properties and important nutritional values, is one of the most economically important fruit crops worldwide. The availability of grapevine genome made it possible and feasible to examine and identify gene families (Boss et al., 1996; Velasco et al., 2007). Although CYP superfamily genes have important regulatory roles in plant growth and development, as well as abiotic/biotic stress, a systematic report on the genome-wide characterization of *VvCYPs* is still lacking in grapevine. Hence, we performed a comprehensive bioinformatics characterization and expression analysis of the *VvCYP* superfamily in grapevine. The present study involved the identification of putative *VvCYPs via* genome-wide searches, the investigation of their phylogenetic relationships, chromosomal distribution, exon–intron organizations, promoter *cis*-acting element analysis, motif pattern, and syntenic analysis. In addition, we also analyzed the expression profiles of the *VvCYP* superfamily in grapevine by RNA-seq for identifying pivotal *VvCYPs* that participated in grapevine growth and development and further validated selected target *VvCYP* genes by qRT-PCR. Additionally, some *VvCYPs* were strongly modulated by ABA treatment, suggesting these *VvCYPs* have important and diverse regulatory roles in responding to ABA. The findings of the current investigations will assist in better comprehending the classification and potential biological functions of *VvCYPs* and pave the way for further functional verification of these *VvCYPs* and are helpful to grapevine molecular breeding.

## Materials and Methods

### Plant Materials, Growth Conditions and ABA Treatments

The grapevine (the hybrid of *V. vinifera* and *V. labrusca* cv. ‘Jumeigui') plants, planted at the farm of Nanjing Agricultural University (Nanjing, China) under standard field conditions, were used as experimental materials. Three biological replicates, each consisting of three clusters, were sampled at each sampling date. The developmental stages were defined in terms of the criterion containing the color, size, sugar content, and softening degree of the berries (Boss et al., 1996). The berry samples were harvested at five development stages, including small green berry (SGB), big green berry (BGB), veraison berry (VB), postveraison berry (PVB), and ripening berry (RB). Additionally, initial flowering (IF), full flowering (FF), young stems (YS), roots (Ro), buds (Bu), young leaves (YL), medium leaves (ML), old leaves (OL), and tendrils (Te) were also collected. Five-year-old uniform grapevine plants were chosen for the ABA treatments. ABA treatments were performed by spraying the grape berry before veraison with a solution containing 50 ppm and 150 ppm ABA, while the control berries were only treated with distilled water. The berry was collected at veraison, postveraison, and ripening. For making one sample, nine berries were collected from three different grapevines with respect to each sampling point and treatment. All of the samples were collected in triplicate and then immediately frozen under liquid nitrogen and kept in a refrigerator at −80°C until further analysis.

### Total RNA Extraction and cDNA Library Construction

The extraction of total RNA was carried out from grapevine samples by following the cetyltrimethylammonium bromide method (Jiu et al., 2015; Jiu et al., 2016). The concentration of total RNA was evaluated using NanoDrop (Thermo Fisher Scientific Inc., USA), and OD_260/280_ ratios were close to 2.0, and OD_260/230_ ratios were >2.0 for all the samples. RNA integrity was also assessed using standard denaturing agarose gel electrophoresis. The samples of total RNA were digested with DNase I (TaKaRa, Japan) against genomic DNA contamination. Then, the manufacturer's protocol was followed for the construction of cDNA libraries with 1.0 μg of total RNA samples (PrimeScriptTM First Strand cDNA synthesis kit, TaKaRa, Japan).

### Mining of *VvCYPs* Superfamily in Grapevine

The sequence of grapevine genome was downloaded from the National Center for Biotechnology Information website (NCBI, ftp://ftp.ncbi.nlm.nih.gov/genomes/Vitis_vinifera/). To identify all *VvCYP* genes in the grapevine, a local BLAST search against the grapevine genome database using 245 known *AtCYPs* acquired by Cytochrome P450 Homepage (http://drnelson.uthsc.edu/CytochromeP450.html) was conducted, setting the threshold of the *e*-value as 1e-10 to confirm the detection of all potential CYP genes in the *Vitis* genome database (http://genomes.cribi.unipd.it/grape/index.php).

Additionally, we also used the seed file of P450 domain (PF00067) from the Pfam online software (http://pfam.xfam.org/) to acquire the Hidden Markov Model (HMM) sequences, then we performed HMM searches using HMMER3 software against the *V. vinifera* protein sequences. As recommended by the HMMER3 user's guide, an *e*-value threshold of 0.1 was performed in these searches (Finn et al., 2011). Subsequently, each candidate VvCYP was used to further confirm the Pfam database. To eliminate repetitive genes, all potential *VvCYPs* were aligned using the DNAMAN5.0 software and manually collated. All the nonoverlapping *VvCYPs* were used for further analysis.

The theoretical isoelectric points (*p*I) and molecular weights (MW) of all members of the grape *VvCYPs* were calculated using ExPASy (http://expasy.org/). The number of transmembrane domains was predicted by the TMHMM Server v. 2.0. (http://www.cbs.dtu.dk/services/TMHMM/). Additionally, the estimation of subcellular localizations of all identified VvCYPs was completed by WoLF PSORT Prediction PSORT II (http://wolfpsort.org/) and TargetP (http://www.cbs.dtu.dk/services/TargetP) software (Emanuelsson et al., 2000).

### Multiple Sequence Alignments and Phylogenetic Analysis

Multiple alignments were performed on the amino acid (AA) sequences of VvCYP proteins in *V*. *vinifera* genomes utilizing the CLUSTALW software with default parameters. Subsequently, MEGA7.0 program was employed to construct a phylogenetic tree based on the alignments using the Neighbor-Joining (NJ) method and bootstrap tests were replicated 1,000 times, which were performed using the *p*-distance model (Ogrodzki and Forsythe, 2015).

### Conserved Motifs and Exon–Intron Organization of *VvCYP* Genes

The AA sequences of 236 VvCYPs were analyzed using the MEME program (http://meme-suite.org/) with the following parameters (optimum width, 15–60; number of repetitions, any; and maximum number of motifs, 15) to identify the conserved motifs (Bailey and Elkan, 1994). The conserved motifs of VvCYPs were confirmed by InterPro software (http://www.ebi.ac.uk/interpro/). The gene structure display server 2.0 (GSDS, http://gsds.cbi.pku.edu.cn ) was employed to display exon–intron organizations of 236 *VvCYPs* (Hu et al., 2014).

### Putative Promoter *Cis*-Acting Element Analysis

The nucleotide sequences of the *VvCYP* superfamily were obtained from the grapevine genome database (http://genomes.cribi.unipd.it/grape/index.php) in this study. The upstream 1,500-bp region from the start codon for all *VvCYPs* was regarded as the promoter sequence (Jiu et al., 2018). The putative *cis*-acting elements of *VvCYP* promoters were identified using PlantCARE online software (http://bioinformatics.psb.ugent.be/webtools/plantcare/html/, Lescot, 2002). The putative *cis*-acting elements involved in phytohormone responses, the regulation of plant growth and development, as well as biotic and abiotic stress responses were summarized.

### Chromosomal Localization and Collinearity Analysis

A total of 236 *VvCYPs* were mapped to the grapevine chromosomes by analyzing their chromosomal localization. The information of chromosomal localization is available at the grapevine genome database (http://genomes.cribi.unipd.it/grape/index.php). The duplication events in the grapevine genome were acquired using the MCscanX software. For syntenic analysis, synteny blocks among the grapevine genome were obtained from the PGDD website (http://chibba.agtec.uga.edu/duplication/index/downloads) and visualized by the Circos software (http://circos.ca/) (Krzywinski et al., 2009).

### Expression Analysis of *VvCYPs*

The expression values normalized in numerous organs and tissues were obtained from the RNA-sequencing database (Fasoli et al., 2012). The primers used for amplifying 24 *VvCYP* genes, designed by using Primer-BLAST online program (https://www.ncbi.nlm.nih.gov/tools/primer-blast/index.cgi? LINK_LOC = BlastHome), are listed in **Table S1**. Polymerase chain reaction (PCR) was used for screening all primer pairs. The expression levels were detected by qRT-PCR assay using a Bio-Rad System (Bio-Rad, CA, USA). Each PCR mixture (20 µl) consisted of 2×TB Green™ Premix Ex Taq™ II (10 µl), 1:10 diluted cDNA (1 µl), each primer (0.4 µl), and RNase-free water (8.2 µl). All reactions were run in 96-well plates, and each cDNA was analyzed in triplicate. The condition of the qRT-PCR was as follows: preincubation (95°C for 30 s) and then 40 cycles (95°C for 5 s, 60°C for 30 s). To detect the relative fold differences for each gene in each experiment, the Ct value of the genes was normalized to the Ct value for the reference genes, and the relative expression levels were calculated using the formula 2^−ΔΔCT^. *KyActin1* was used as the internal reference control. The lowest expression levels of the samples were manually set to 1.

### Subcellular Localization

The full-length cDNAs of the *VvCYP710A1* and *VvCYP51G1a* were amplified using the primers (**Table S1**) and cloned into the binary vector PHB including two cauliflower mosaic virus (CaMV) 35S promoter, a translation enhancer, and a GFP fluorescent protein tag, respectively, to generate two fusion constructs (p35S-*VvCYP710A1*-GFP and p35S-*VvCYP51G1a*-GFP). After identifying two sequences, the fusion constructs and the control vector (PHB) were transformed into *A. tumefaciens* GV3101 strains and subsequently agroinfiltrated into the leaves of 3 to 5-week-old *Nicotiana benthamiana* plants. Localization of fluorescent proteins was observed 3–7 days after infiltration, the period when GFP fluorescence was optimal, by using a confocal laser scanning microscope (Zeiss LSM 780, Germany) according to the manufacturer's instructions.

### Statistical Analysis

The experiment was arranged in a completely randomized design (CRD) with three replications in this study and collected data were statistically analyzed by SAS computer software (SAS Version 9.2, Institute). Analysis of variance (ANOVA) was used to determine the overall statistical significance of the data at level of *P* < 0.05 and data were represented as average ± STDEV (n = 3).

## Results

### Identification and Analysis of Full-Length *VvCYPs* in Grapevine

The availability of grapevine genome sequences provides the sources in the genome-wide identification of the grapevine *VvCYP* superfamily (Jaillon et al., 2007). After bioinformatics analyses, a total number of 236 *VvCYP* genes with conserved P450 domain have been identified and characterized in this study (**Table S2**). Here, we designated the *VvCYP* genes in grapevine according to the *Arabidopsis* CYP genes with the highest sequence similarity and following the classification of gene terminology used in the *Arabidopsis*. All *VvCYPs* were divided into 46 families and grouped into A-type (27 families) and non-A type (19 families) including 102 and 134 *VvCYPs*, respectively. Each of the 13 families (VvCYP701, VvCYP703, VvCYP712, VvCYP83, VvCYP98, VvCYP709, VvCYP715, VvCYP735, VvCYP702, VvCYP720, VvCYP724, VvCYP710, and VvCYP711) has only a single gene, whereas VvCYP71 with 25 members is the largest family, followed by VvCYP72 family with 21 members. The AA sequence lengths of the 236 identified VvCYP proteins ranged from 269 to 579 with an average of 484.40 AA. The molecular weights (Mw) of these VvCYP proteins ranged from 23.48 kDa (VvCYP72A10c) to 144.19 kDa (VvCYP79A2e), and the isoelectric points (*p*I) ranged from 4.92 (VvCYP74B2) to 5.31 (VvCYP72A10c). Other characteristics of the *VvCYPs*, including the chromosome location, number of transmembrane domains, gene duplication, conserved motif, and prediction of subcellular localization are also shown in **Table S2**.

### Phylogenetic Analysis of *VvCYP* Genes in Grapevine

**Figure 1:**
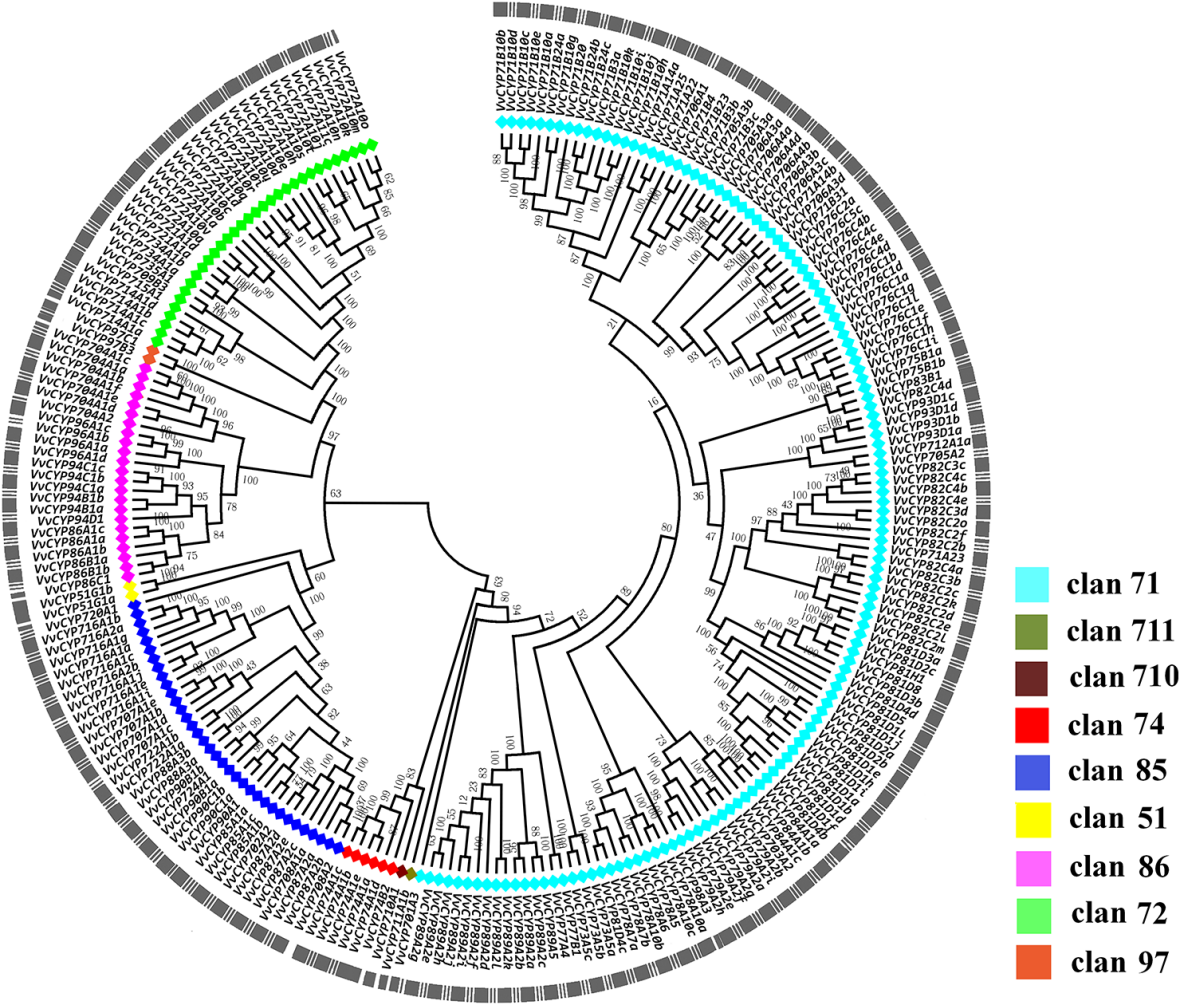
The phylogenetic tree of VvCYPs superfamily in grapevine. The unrooted phylogenetic tree was constructed using the full-length AA sequences of VvCYPs using MEGA7.0 by the Neighbor-Joining (NJ) method with 1000 bootstrap replicates. The entire family of *VvCYPs* is shown for each clan with different color: aquamarine, clan 71; olivedrab, clan 711; prunosus, clan 710; red, clan 74; royalblue, clan 85; yellow, clan 51; violet, clan 86; springgreen, clan 72; salmon, clan 97.

To elucidate the phylogenetic relationships among the members of the *VvCYP* superfamily, an unrooted tree was constructed from an alignment of their AA sequences and produced in MEGA 7.0 software by the NJ method. Based on the phylogenetic analysis, *VvCYP*s were grouped into nine clans (clan 51, clan 71, clan 72, clan 74, clan 85, clan 86, clan 97, clan 710, and clan 711) with well-supported bootstrap values (**Figure 1**). There were one family and two *VvCYPs* in clan 51, 19 families and 134 *VvCYPs* in clan 71, seven families and 32 *VvCYPs* in clan 72, one family and six *VvCYPs* in clan 74, 11 families and 34 *VvCYPs* in clan 85, four families and 23 *VvCYPs* in clan 86, one family and two *VvCYPs* in clan 97, one family and one *VvCYP* in clan 710, one family and one *VvCYP* in clan 711. The three largest clans (in descending order) were clan 71, clan 85 and clan 72. Furthermore, the nine clans were classified into four clusters. Clan 74 was thought to be a special one, and clan 51 and clan 85 were classified into a cluster. Clan 72, clan 86, and clan 97 were gathered in one cluster, and clan 710, clan 711, and clan 71 were closely grouped together to form another cluster.

**Figure 2:**
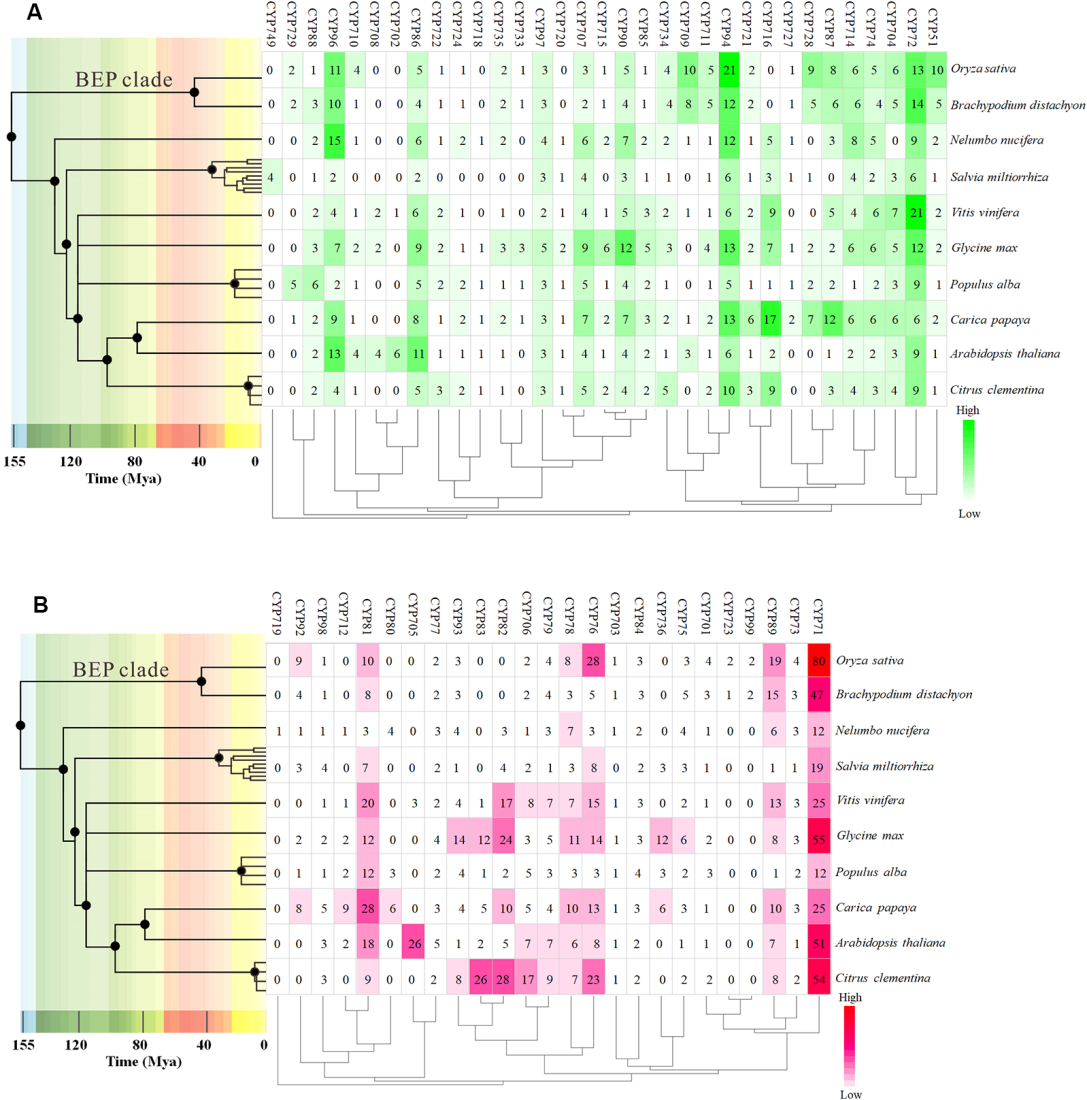
The distribution of CYP family sizes in whole-genome for 10 species. **(A)** A-type CYP families. **(B)** Non-A type CYP families. The time tree generated using TIMETREE web resource (http://www.timetree.org/) revealed the clock-like speciation and diversification of 10 species. The four species, *Nelumbo nucifera*, *Salvia miltiorrhiza*, *Populus alba*, and *Carica Papaya*, were instead by other species of the same genus on the time tree.

To further investigate the evolution and divergence of the *VvCYP* superfamily in grapevine, comprehensive comparisons among *V. vinifera*, *A. thaliana*, *O. sativa*, *Populus alba*, *Brachypodium distachyon*, *Carica papaya*, *Salvia miltiorrhiza*, *Citrus clementina*, *Nelumbo nucifera*, and *Glycine max* were performed. Some CYP subfamilies only present in dicots, such as CYP716, CYP720, and CYP82, whereas CYP99 and CYP723 only exist in monocots (**Figure 2**). Many CYP families were found in ten species, for instance, CYP88, CYP94, CYP86, CYP71, and CYP73. However, some families were only present in one of ten species, such as CYP749 in *S. miltiorrhiza*, CYP719 in *N. nucifera*. It's worth noting that all defined grapevine VvCYP subfamilies emerged after divergence of grapevine and *N. nucifera*. The gene number-based hierarchical cluster of the CYP subfamilies of different plants exhibited that the loss of the CYP family seems to be limited to single species or single taxa. The expansion of the CYP families can be observed in the CYP71, CYP72, CYP76, CYP81, CYP89, and CYP94 families (**Figure 2**). Particularly, the members of the CYP94 family are closely linked with the metabolism of aliphatic acid, and the CYP72 family with multiple functions in *V. vinifera* are more abundant than those in the other species.

### Conserved Motifs, Chromosomal Locations, Exon–Intron Organizations of *VvCYP* Genes

**Figure 3:**
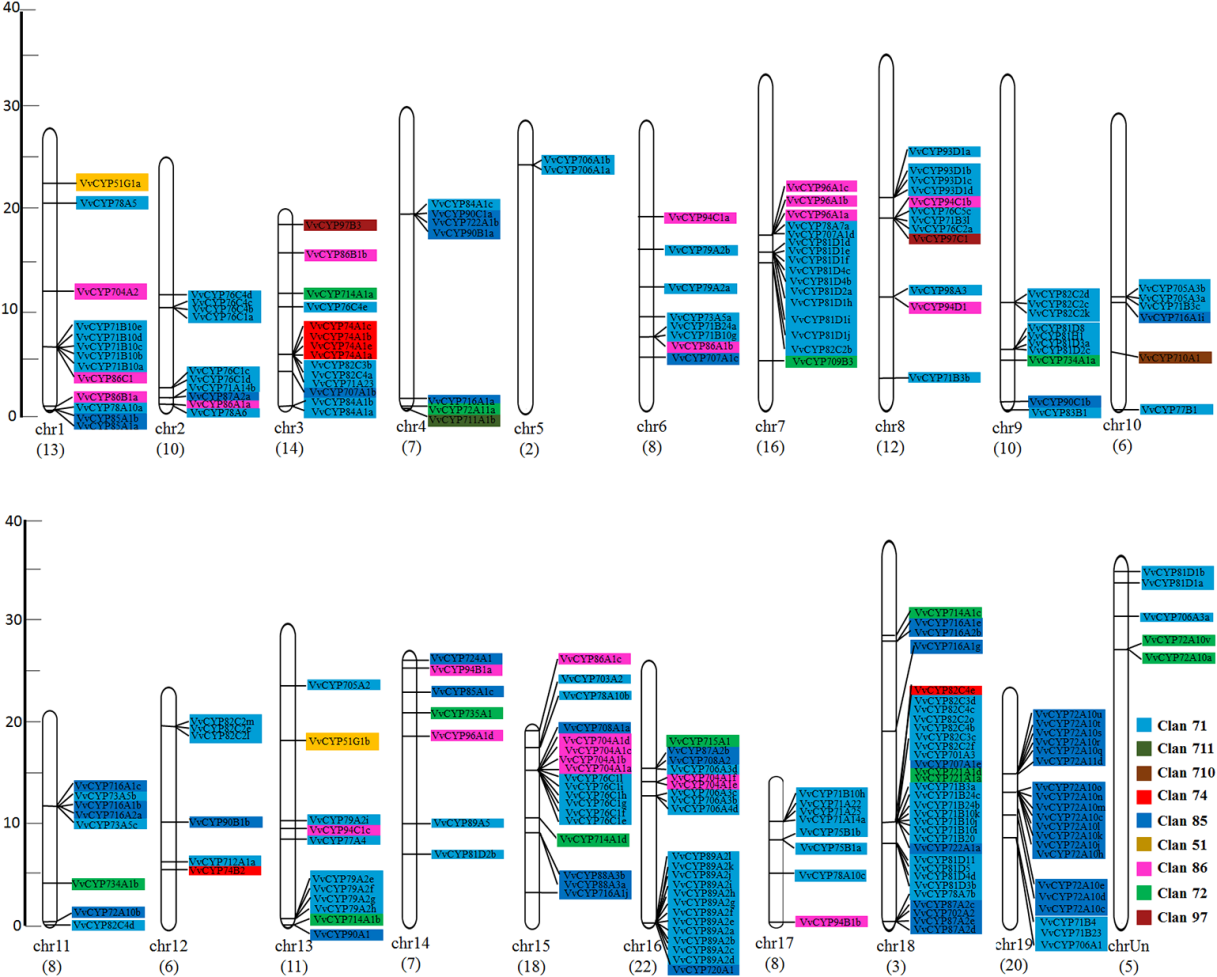
The chromosomal location of *VvCYPs* from *V. vinifera*. The scale represents 40 Mb chromosomal distance. The *VvCYP* numbers are marked on the bottom of chromosomes. The numbers on the left side of the bars designated the approximate physical position of the first exon of corresponding *VvCYPs* on grapevine chromosomes. The *VvCYPs* of each clan were shown with different background color: aquamarine, clan 71; olivedrab, clan 711; prunosus, clan 710; red, clan 74; royalblue, clan 85; yellow, clan 51; violet, clan 86; springgreen, clan 72; salmon, clan 97.

**Figure 4:**
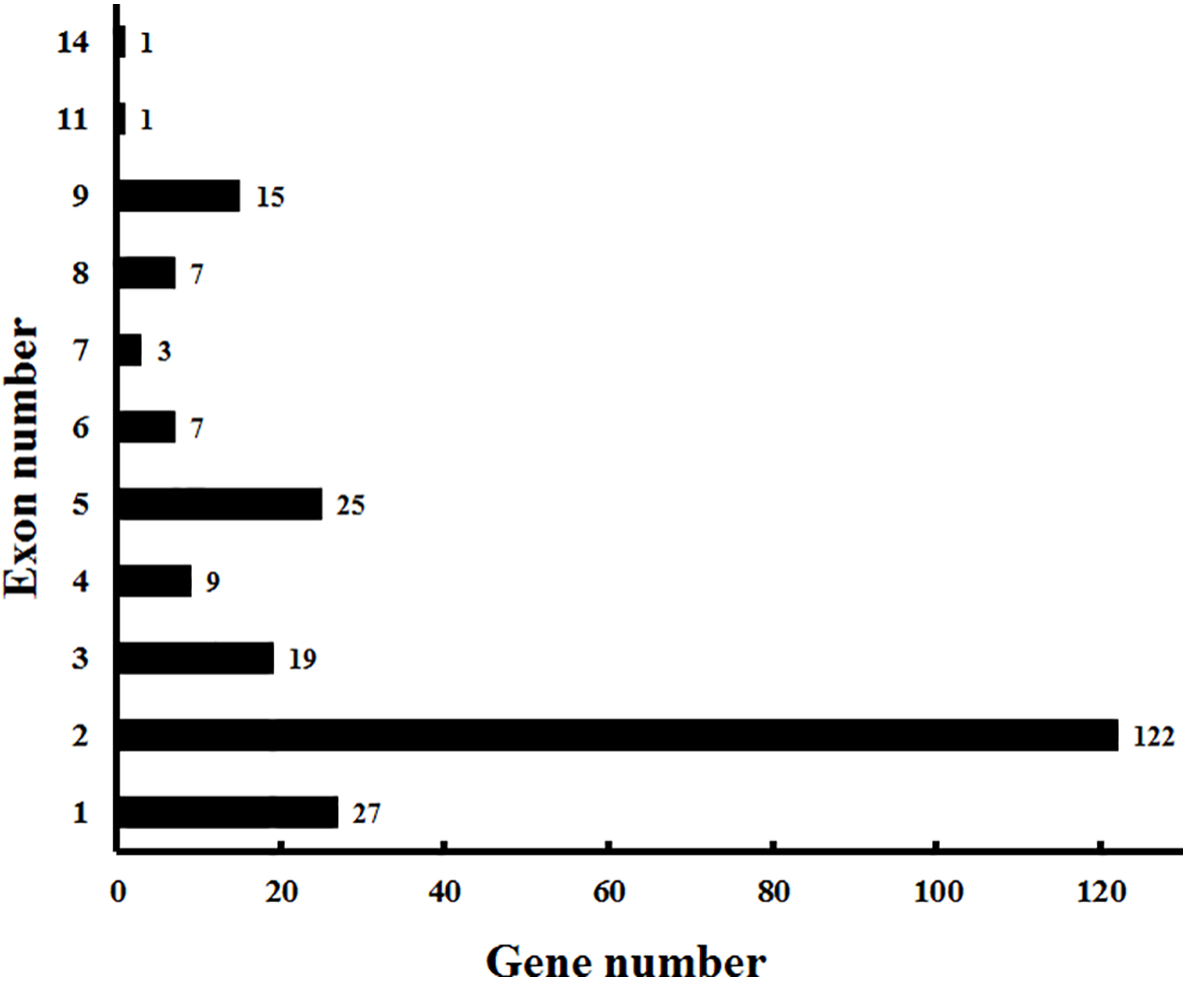
Exon distribution of *VvCYPs* in grapevine. The exon information of *VvCYPs* was obtained from the *Vitis* genome database (http://genomes.cribi.unipd.it/grape/index.php).

In addition to the predictions of subcellular localizations and the analysis of transmembrane domains, the evaluation of conservative motifs is one of the important means for the functional prediction of VvCYP proteins. In this study, a sum of 15 conserved motifs (motifs 1–15) were predicted using MEME online software (**Figure S1**). Several motifs were found in most VvCYP proteins, such as motifs 1, 2, 3, 4, and 7. However, some are only present in a specific clan, such as motifs 9 and 10 in clan 71. Additionally, clan 71 contained motifs 1–15 and motif 2 existed in all VvCYP members of clan 71, whereas clan 711 only included one member, VvCYP711A1b had motifs 1, 3, and 7 (**Figure S2** and **Table S3**). Most of VvCYP proteins within the same clan exhibited similar motif components while a high discrepancy was observed among different clans, indicating that the VvCYP members within the same family may undertake semblable functions, and that some motifs may play a vital role in the family-specific functions. The chromosomal location of each *VvCYP* gene in the grapevine genome is shown in **Figure 3**. A total of 236 *VvCYPs* are unevenly distributed on 19 chromosomes. Out of the 236 *VvCYPs*, 33 were mapped onto chromosome (chr)18, followed by 22 on chr16, whereas only 2 *VvCYPs* were located on chr5. The *VvCYPs* with relatively high densities were detected on the bottom and middle arms of the chromosome. To better comprehend the structure of *VvCYPs*, the exon–intron organizations were analyzed by the alignment of cDNA sequences and corresponding genomic DNA sequences. As shown in **Figure S3**, 26 out of 236 *VvCYPs* had no intron. As **Figure 4** demonstrates, grapevine VvCYP families possessed different exon numbers, ranging from one to 14. *VvCYP97B3*, which is a member of clan 86, contains 14 exons. Most members within the identical family showed similar exon–intron distribution characteristics in accordance with exon length and number. For example, 15 *VvCYPs* contain nine exons, 14 of which belong to the CYP85 family. 25 *VvCYPs* contain four exons, 24 of which belong to the CYP72 family. *VvCYPs* in the same families showed great consistence with exon length and number. Only a few of *VvCYPs* had no typical genetic structure. For example, *VvCYP81D3b*, *VvCYP82C2o*, *VvCYP73A5a*, *VvCYP78A5,* and *VvCYP706A4d* in clan 71, *VvCYP72A10d, VvCYP72A10e,* and *VvCYP72A10q* in clan 72 had a long intron and short exon organization compared with the other members within the same family. The majority of them share two (51.7%, 122/236), one (11.4%, 27/236), and five (10.6%, 25/236) exons. At the DNA level, *VvCYP97B3* with 28 kb genomic sequences was the longest *VvCYP* gene identified in this study. The great change in structures of *VvCYPs* could demonstrate that the grape genome had changed significantly during its long evolutionary history. The coherence of the exon/intron organizations and motif structures further supported the close evolutionary relationships of the grapevine *VvCYP* superfamily as well as the reliability of the phylogenetic analysis.

### Gene Duplication and Syntenic Analysis of the *VvCYP* Genes

**Figure 5:**
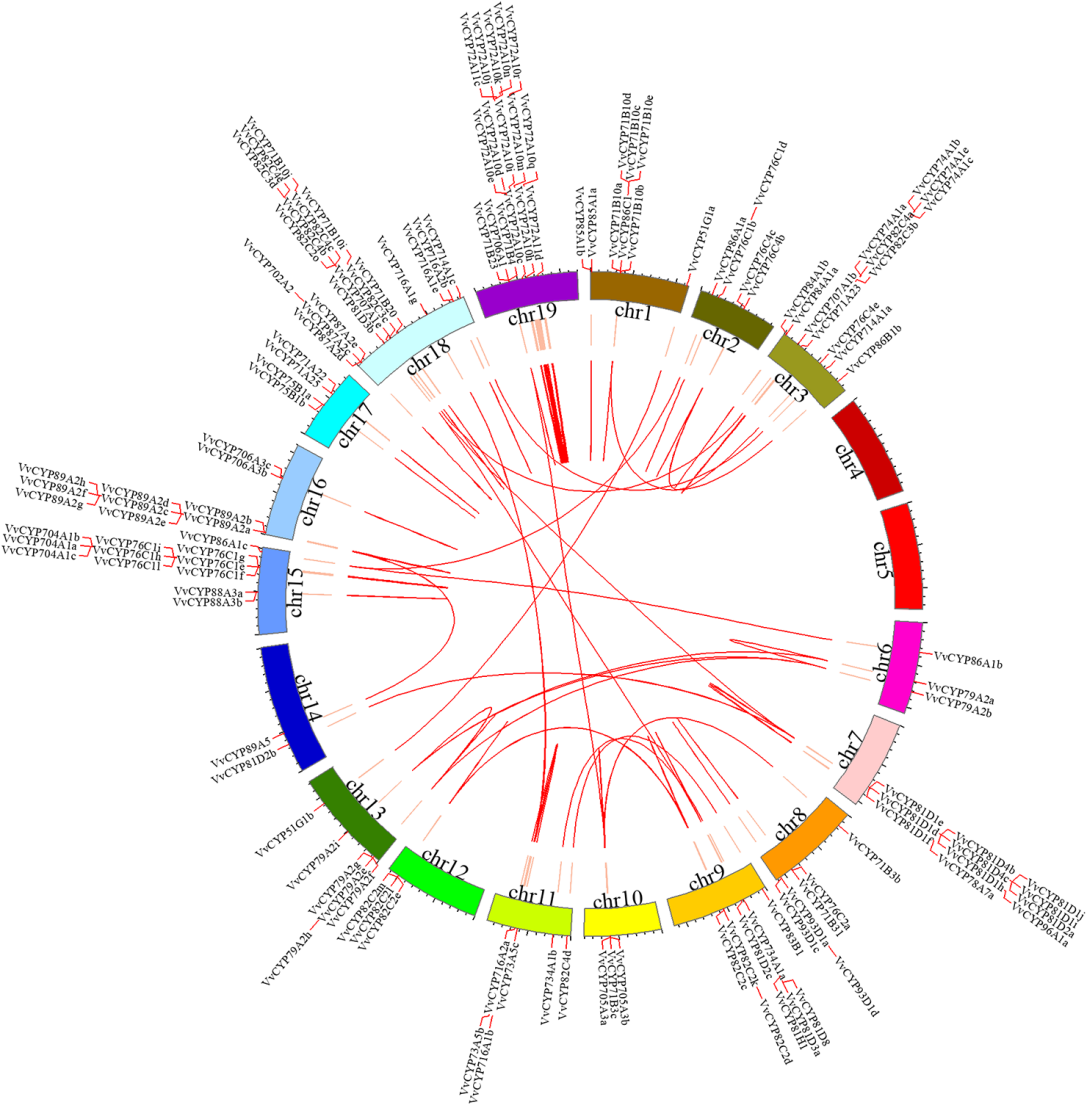
Syntenic block of the *VvCYPs* superfamily. Red lines mark the *VvCYP* gene positions on chromosomes. Chromosomal locations were determined according to Grape Genome Database (http://genomes.cribi.unipd.it/grape/index.php). Red curves in circle represented the syntenic relationships among *VvCYP* genes. The map was obtained using Circos software.

**Figure 6:**
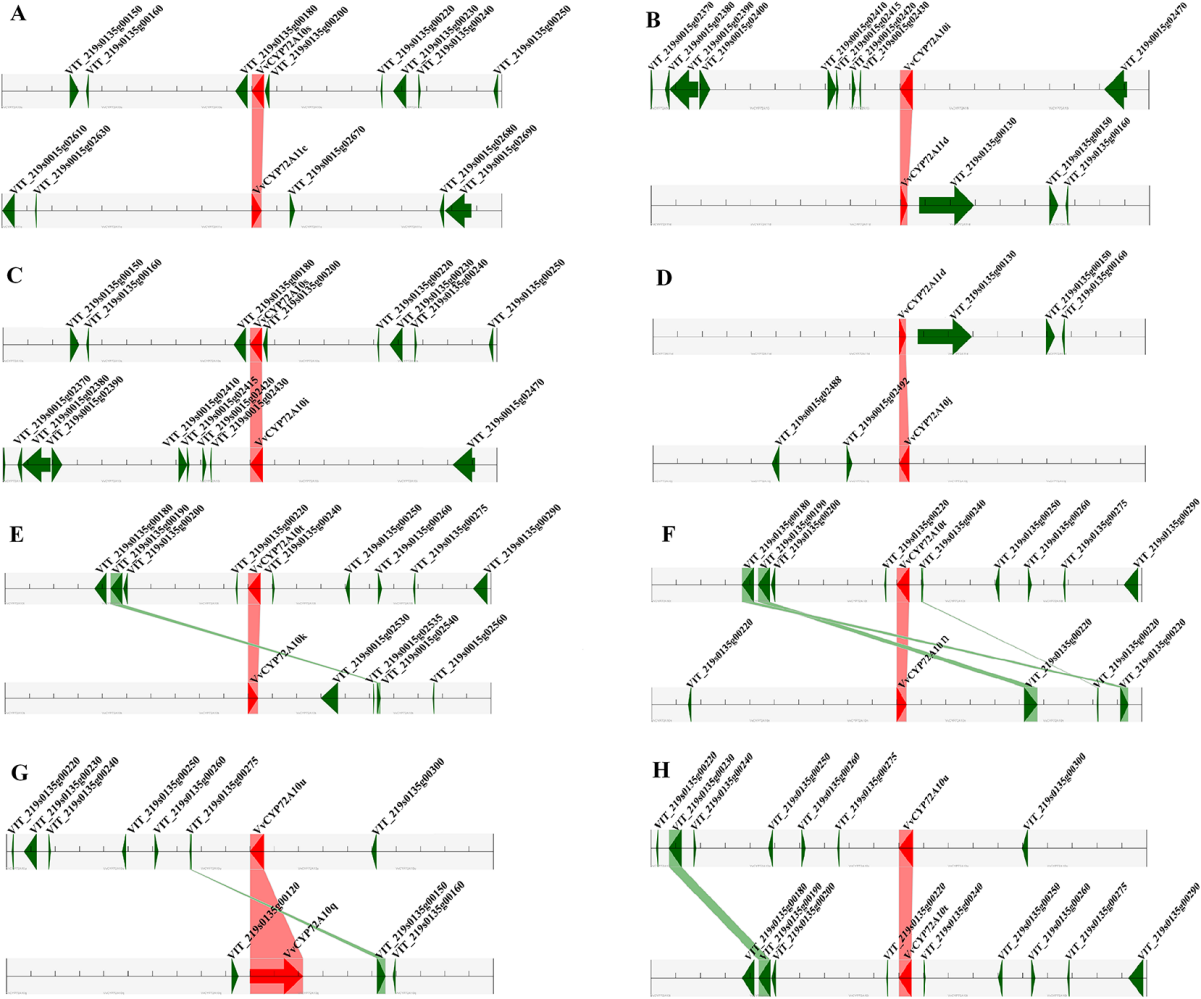
Segmental duplication between members of the *VvCYPs* subfamily. **(A)** *VvCYP72A10s* (VIT_219s0135g00190.1) and *VvCYP72A11c* (VIT_219s0015g02660.1), **(B)** *VvCYP72A10i* (VIT_219s0015g02440.2) and *VvCYP72A11d* (VIT_219s0135g00120.1), **(C)** *VvCYP72A10s* (VIT_219s0135g00190.1) and *VvCYP72A10i* (VIT_219s0015g02440.2), **(D)** *VvCYP72A11d* (VIT_219s0135g00120.1) and *VvCYP72A10j* (VIT_219s0015g02500.1), **(E)** *VvCYP72A10t* (VIT_219s0135g00230.1) and *VvCYP72A10k* (VIT_219s0015g02520.1), **(F)** *VvCYP72A10t* (VIT_219s0135g00230.1) and *VvCYP72A10n* (VIT_219s0015g02900.1), **(G)** *VvCYP72A10u* (VIT_219s0135g00290.1) and *VvCYP72A10q* (VIT_219s0135g00130.1), **(H)** *VvCYP72A10u* (VIT_219s0135g00290.1) and *VvCYP72A10t* (VIT_219s0135g00230.1). The figure shows a region of 100 kb on each side flanking the *VvCYP* genes. Homologous gene pairs are connected with bands. Chromosome segments are indicated by black horizontal lines, and a grey line with arrowhead represents a gene. The *VvCYP* genes are shown in red, and homologous genes are linked by the pink line, other genes are shown in green, and homologous genes are linked by the green line.

For the gene family expansion and evolution of novel functions, gene duplication and divergence are essential steps in the plant genome. *Vitis* spp. has suffered from whole-genome duplications during its generative history (Jaillon et al., 2007). Moreover, several gene duplication events including tandem duplication, WGD/segmental, and rearrangements at the chromosomal and gene levels, drive the evolution of gene families encoding proteins (Maher et al., 2006). To evaluate the effect of duplications on the *VvCYP* superfamily, we firstly analyzed the origins of duplicate genes for the *VvCYP* superfamily in the grapevine genome utilizing the MCScanX software. Each member of the *VvCYPs* was allocated to one of five duplication events (tandem, dispersed, singleton, WGD/segmental, and proximal). The results showed that 48.31% (114) of the *VvCYPs* in grapevine were duplicated from tandem event, compared with 19.92% (47) from singleton, and 16.10% (38) from proximal event, 11.02% (26) from dispersed event, and 4.66% (11) from WGD/segmental event (**Table S2**). The results demonstrated that tandem duplication was the major driving force for the augmentation of the *VvCYP* superfamily (**Table S2**). In addition, the chromosome distribution patterns of *VvCYPs* powerfully showed that tandem duplication contributed to the expansion of *VvCYPs* in *V. vinifera*. To explore further the probable evolutionary mechanisms of the *VvCYP* superfamily, we used a method similar to that used for the Plant Genome Duplication Database (PGDD) to identify synteny blocks across the whole grapevine genome (**Figure 5**). A sum of 41 segmentally duplicated gene pairs were detected in the *VvCYP* superfamily. As **Figure 6** shows, eight segmentally duplicated gene pairs within grapevine genome included: *VvCYP72A10s* and *VvCYP72A11c*, *VvCYP72A10i* and *VvCYP72A11d*, *VvCYP72A10s* and *VvCYP72A10i*, *VvCYP72A11d* and *VvCYP72A10j*, *VvCYP72A10t* and *VvCYP72A10k*, *VvCYP72A10t* and *VvCYP72A10n*, *VvCYP72A10u* and *VvCYP72A10q*, *VvCYP72A10u* and *VvCYP72A10t*.

### Identification of the Putative *Cis*-Acting Elements in the Promoter of *VvCYP* Genes

The evaluation of *cis*-elements in promoters is a key factor for comprehending transcriptional regulation and gene function. To recognize the putative *cis*-regulatory elements of *VvCYPs*, the promoter sequences of 236 *VvCYPs* were searched in the *Vitis* genomic database (http://genomes.cribi.unipd.it/grape/index.php). In this study, a 1,500-bp was considered as a promotor region for 221 *VvCYPs*, whereas, a <1,500 bp promoter sequence for the *VvCYP705A2*, *VvCYP712A1a*, *VvCYP81D3b*, *VvCYP81D1l*, *VvCYP81D4d*, *VvCYP82C4c*, *VvCYP71A25*, *VvCYP76C1d*, *VvCYP76C5c*, *VvCYP76C2a*, *VvCYP82C3c*, *VvCYP89A2a*, *VvCYP706A4a*, *VvCYP72A10s*, and *VvCYP707A1d* was also identified because of the existence of another gene situated at the <1,500 bp upstream (**Data Sheet 1**). A total of 107 putative *cis*-elements involved in plant hormone [*e.g.*, auxin, ABA, MeJA, ethylene, gibberellin (GA), and salicylic acid (SA)] responses, biotic and abiotic stress responses, and plant growth and development were identified using the PlantCARE online database. As shown in **Table S4**, the predicted *cis*-elements differed among the 236 *VvCYPs*, and two *cis*-elements (TATA-box and CAAT-box) were the most abundant, which had the largest number in all 236 *VvCYPs*. It is quite interesting to recognize that these unique *cis*-elements (AuxRR-core, AACA motif and 4cl-CMA2b, *etc*.) were only present in one of 236 *VvCYPs*, implying that these gene-specific *cis*-elements might play a special role in regulating some biological processes. Some phytohormone-related *cis*-regulatory elements including the ABA response element (ABRE), the GA-responsive element (P-box), the SA-responsive element (TCA), the auxin-responsive element (TGA-element), the MeJA response element (CGTCA-motif), and the ethylene-responsive element (ERE) were found in the promoters of 144, 49, 70, 48, 115, and 172 *VvCYPs*, respectively. A mass of phytohormone-responsive elements were recognized in the *VvCYP* promoters, suggesting they could play an important role in the regulation of grape growth and development process. In several *cis*-elements related to plant growth and development, the zein-metabolism regulation element (O_2_-site), the cell growth promotion element (MYC), the circadian regulator element (circadian), the leaf development-related *cis*-regulatory element (HD-Zip1), the seed-specific regulation element (RY-element), the endosperm-specific expression element (GCN4_motif), the flavonoid biosynthetic regulation element (MBSI), and the meristem expression element (CAT-box) were identified in the promoters of 67, 214, 44, 19, 10, 19, 6, and 62 *VvCYPs*, respectively. Additionally, some stress-related *cis*-regulatory elements (*e.g.* DRE core, LTR, and WUN-motif) that are associated with drought, low-temperature, and wound responses, were found in 6 (2.54%), 97 (41.10%), and 46 (19.49%) *VvCYPs*, respectively.

### Tissue-Specific Transcript Accumulation Patterns of *VvCYP* Genes in Grapevine

**Figure 7:**
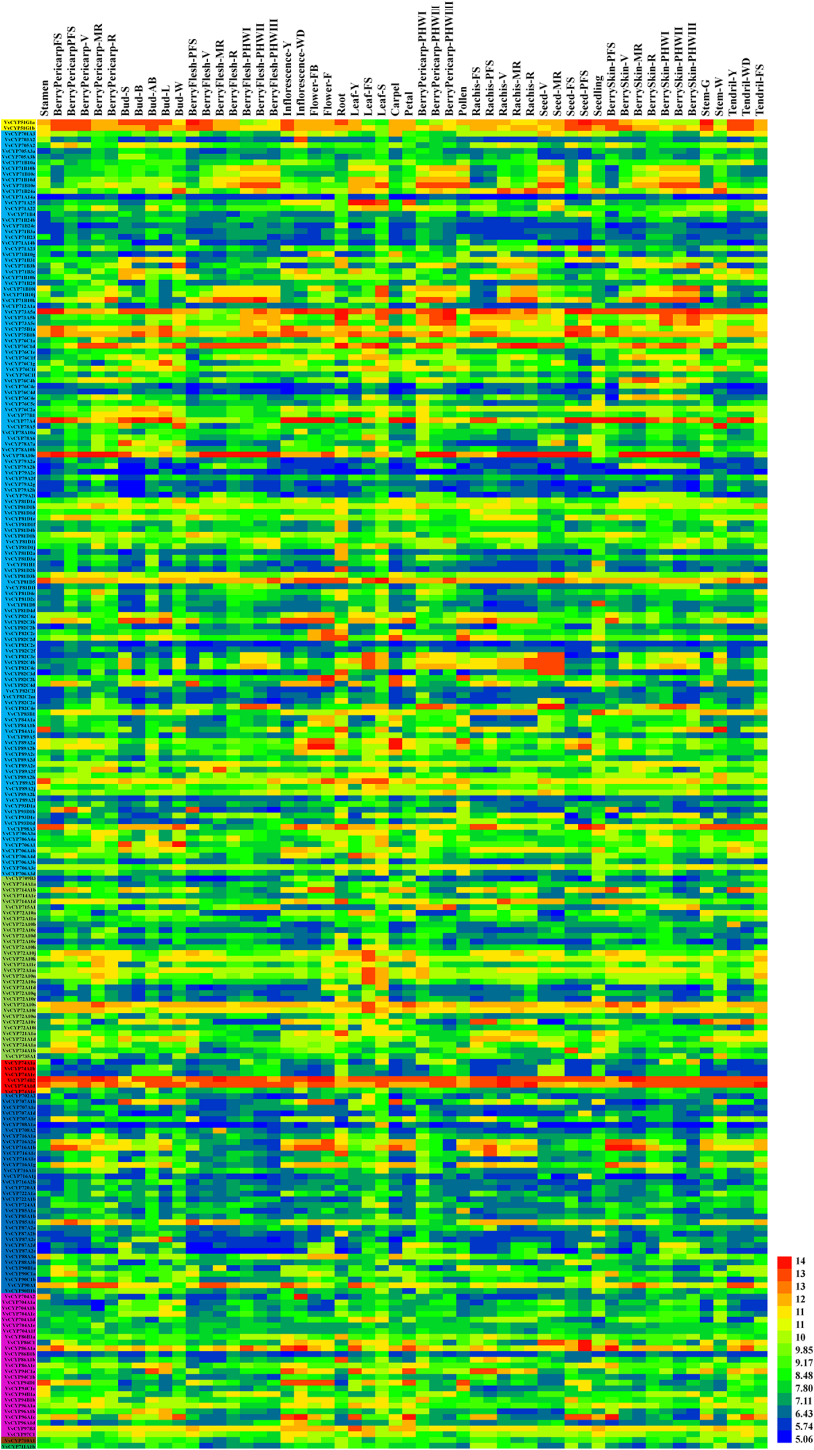
Expression profiles of *VvCYP* genes in the *V. vinifera* cv “Corvina” atlas (GEO Accession: GSE36128). Data were normalized based on the mean expression value of each gene in all tissues (Fasoli et al., 2012). The mean expression values were again normalized using logarithm with the base of 2 using the Heml software. Blue and red boxes show low and high expression levels, respectively, for each gene.

**Figure 8:**
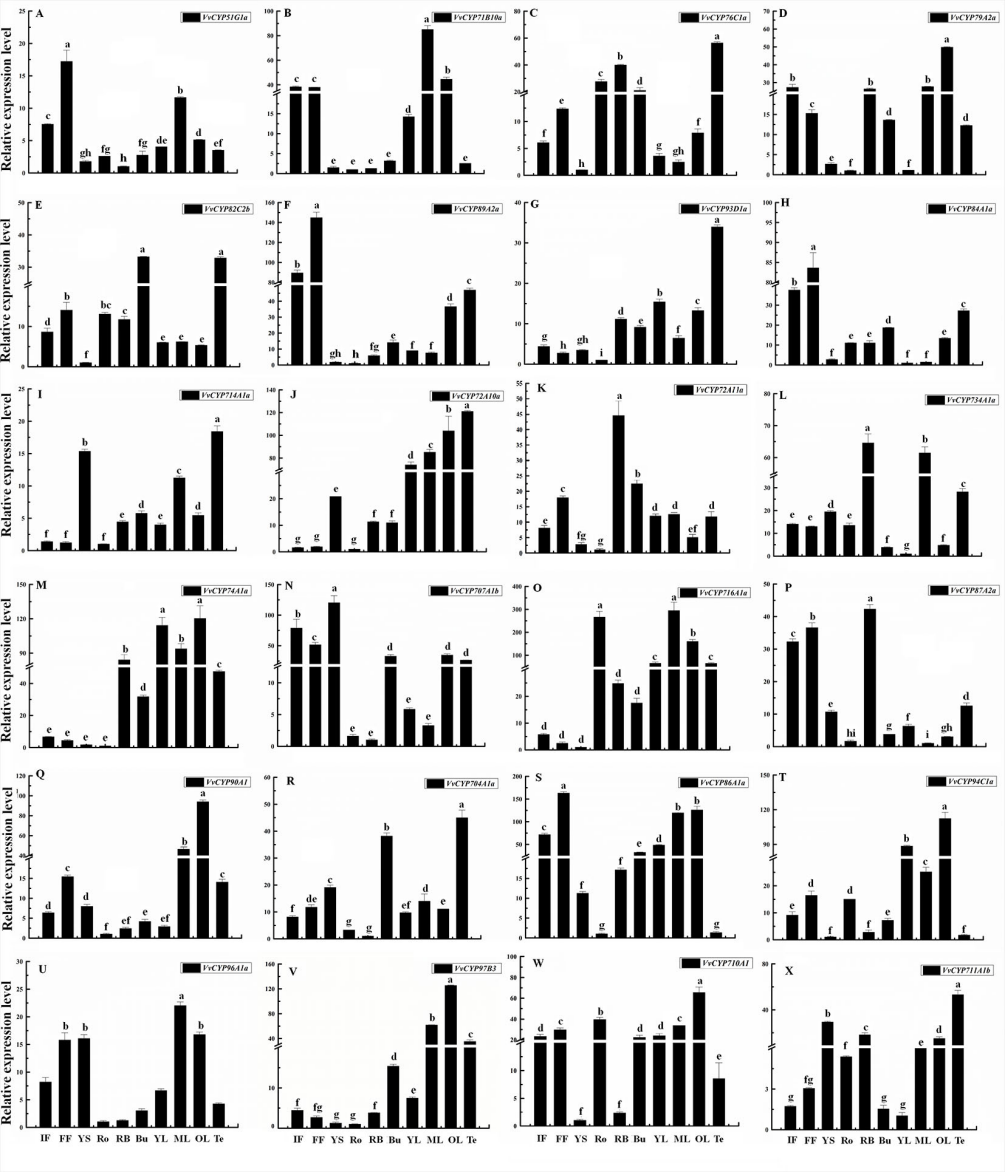
Relative expression levels of *VvCYPs* in different organs of ‘Jumeigui’ grapevine. Values were normalized against the expression data of *KyActin1* and are given as means ± standard deviation among three biological replicates. Different letters indicate significant differences between genes (*p*< 0.05). The expression levels were calculated based on the 2^−△△Ct^ method. Additionally, initial flowering (IF), full flowering (FF), young stems (YS), roots (Ro), buds (Bu), young leaves (YL), medium leaves (ML), old leaves (OL) and tendrils (Te). The expression levels of *VvCYP51G1a* **(A)**, *VvCYP71B10a* **(B)**, *VvCYP76C1a* **(C)**, *VvCYP79A2a* **(D)**, *VvCYP82C2b* **(E)**, *VvCYP89A2a* **(F)**, *VvCYP93D1a* **(G)**, *VvCYP84A1a* **(H)**, *VvCYP714A1a* **(I)**, *VvCYP72A10a* **(J)**, *VvCYP72A11a* **(K)**, *VvCYP734A1a* **(L)**, *VvCYP74A1a* **(M)**, *VvCYP707A1b* **(N)**, *VvCYP716A1a* **(O)**, *VvCYP87A2a* **(P)**, *VvCYP90A1* **(Q)**, *VvCYP704A1a* **(R)**, *VvCYP86A1a* **(S)**, *VvCYP94C1a* **(T)**, *VvCYP96A1a* **(U)**, *VvCYP97B3* **(V)**, *VvCYP710A1* **(W)**, and *VvCYP711A1b* **(X)** in different organs of ‘Jumeigui’ grapevine.

To gain more insight into the role that *VvCYPs* play in grapevine growth and development, the expression patterns of *VvCYPs* were analyzed in different grapevine organs and tissues based on global RNA-sequencing data (Fasoli et al., 2012). Interestingly, VvCYP74B2 and VvCYP81D5 showed consistently high expression in all 54 tissues, whereas VvCYP71A14a displayed lower expression levels in all tested tissues compared with the others (**Figure 7**). Additionally, many gene expression profiling exhibited that diverse temporal, spatial, and tissue-specific expression patterns of *VvCYPs* in all tested tissues, potentially indicating the functional divergence of *VvCYPs*. To further clarify possible functions of *VvCYP* genes in grapevine, the expression levels of selected target *VvCYPs* participating in grapevine growth and development were examined in different tissues including fruits, leaves, roots, buds, stems, tendrils, and flowers (**Figure 8**). The expression levels of various genes in different tissues were unlike, indicating that the functions of *VvCYPs* in different tissues were specific to the grapevine growth and development. *VvCYP76C1a*, *VvCYP82C2b,* and *VvCYP93D1a*, three members of clan 71, were highly expressed in tendrils, displaying that they might play a vital role in the growth and development of tendrils. Meanwhile, seven *VvCYPs* (*VvCYP71B10a*, *VvCYP76C1a*, *VvCYP79A2a*, *VvCYP82C2b*, *VvCYP89A2a*, *VvCYP93D1a*, and *VvCYP84A1a*) belonging to clan 71 are all weakly expressed in young stems. Furthermore, the expression levels of *VvCYP714A1a* and *VvCYP72A10a,* belonging to clan 72, displayed similar tendency in different tissues. In particular, *VvCYP72A11a* was preferentially expressed at high levels in RB, which indicated that it might make a difference in fruit maturation. *VvCYP74A1a,* belonging to clan 74, presented very high expression levels in RB and leaves; however, low expression levels were observed in flowers, roots, and young stems. In four members of clan 85, *VvCYP707A1b* and *VvCYP87A2b* were highly expressed in flowers; whereas *VvCYP716A1a* and *VvCYP90A1* presented very high expression levels in ML and OL, possibly indicating their functional divergence. Additionally, several *VvCYPs* of clan74 displayed more extensive tissue-specific expression patterns; such as, *VvCYP704A1a* was hugely expressed in tendrils and buds; *VvCYP94C1a* displayed high relative transcript levels in YL and OL, *VvCYP96A1a* and *VvCYP97B3* were highly expressed in ML. *VvCYP710A1* showed more widespread but less tissue-specific expression patterns in different tissues, except for YS and RB. These results urged us to research the expression level of *VvCYPs* during different berry developmental stages.

### Transcript Accumulation Patterns of *VvCYPs* During Different Fruit Developmental Stages

**Figure 9:**
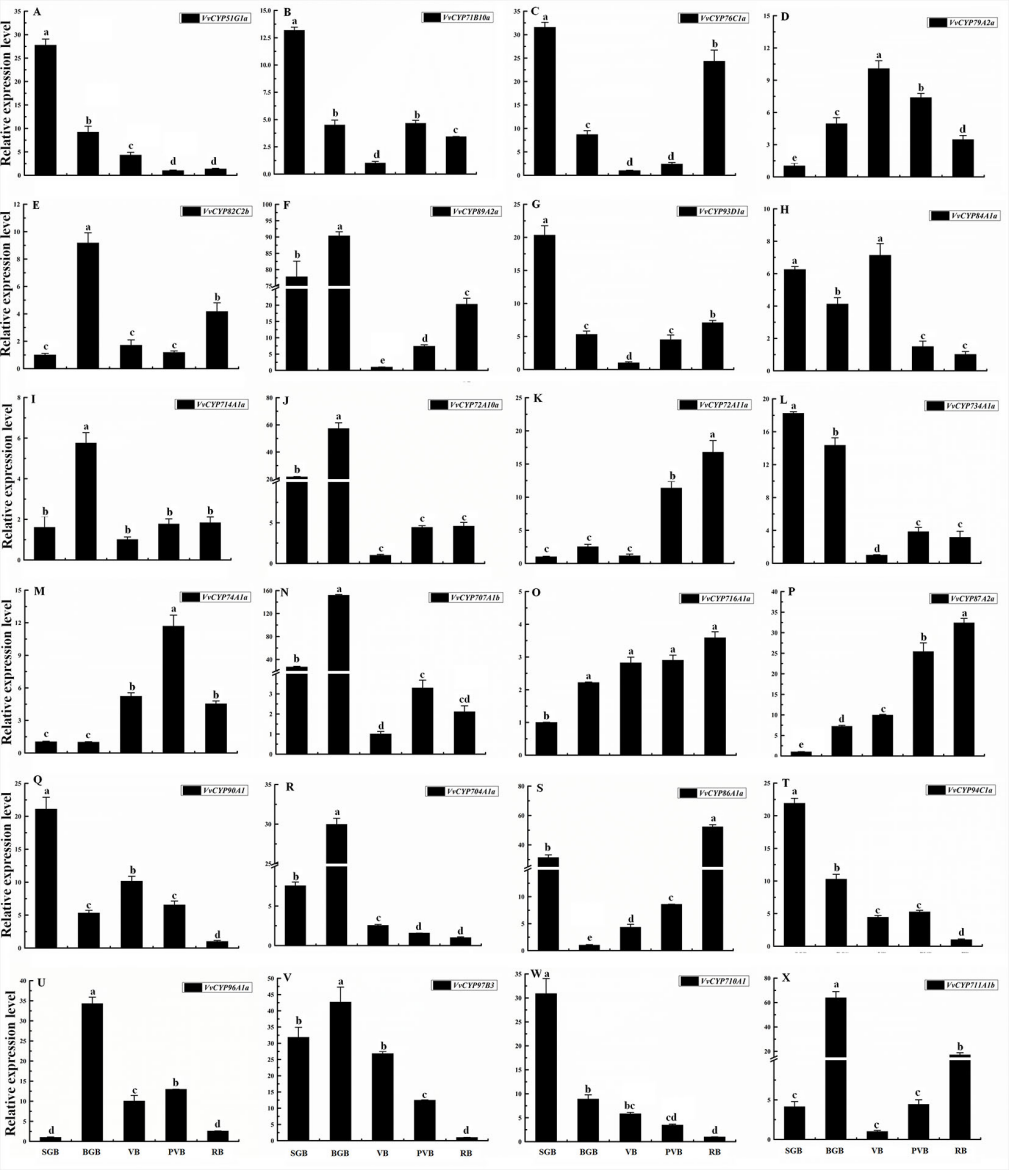
Relative expression levels of *VvCYPs* in different fruit development of ‘Jumeigui’ grapevine. Values were normalized against the expression data of *KyActin1* and are given as means ± standard deviation among three biological replicates. Different letters indicate significant differences between genes (*p*< 0.05). The expression levels were calculated based on the 2 ^−△△Ct^ method small green berry (SGB), big green berry (BGB), veraison berry (VB), post-veraison berry (PVB) and ripening berry (RB). The expression levels of VvCYP51G1a **(A)**, VvCYP71B10a **(B)**, VvCYP76C1a **(C)**, VvCYP79A2a **(D)**, VvCYP82C2b **(E)**, VvCYP89A2a **(F)**, VvCYP93D1a **(G)**, VvCYP84A1a **(H)**, VvCYP714A1a **(I)**, VvCYP72A10a **(J)**, VvCYP72A11a **(K)**, VvCYP734A1a **(L)**, VvCYP74A1a **(M)**, VvCYP707A1b **(N)**, VvCYP716A1a **(O)**, VvCYP87A2a **(P)**, VvCYP90A1 **(Q)**, VvCYP704A1a **(R)**, VvCYP86A1a **(S)**, VvCYP94C1a **(T)**, VvCYP96A1a **(U)**, VvCYP97B3 **(V)**, VvCYP710A1 **(W)**, and VvCYP711A1b **(X)** in different fruit development of ‘Jumeigui’ grapevine.

To illuminate their functions in fruit development, qRT-PCR was employed to explore the expression levels of selected target *VvCYPs* belonging to different subfamilies different fruit developmental stages. As shown in **Figure 9**, the expression levels of six *VvCYPs* (*VvCYP51G1a*, *VvCYP71B10a*, *VvCYP734A1a*, *VvCYP90A1*, *VvCYP94C1a*, and *VvCYP710A1*) were down-regulated toward fruit maturity, which suggested that they might function mainly in the early period of berry development. On the contrary, the transcripts of three genes (*VvCYP72A11a*, *VvCYP716A1a*, and *VvCYP87A2a*) were up-regulated during the ripening process, which indicated that they might play a vital role in fruit maturation. *VvCYP79A2a* transcript was continuously increased from SGB to VB but declined afterward as the fruit matured. *VvCYP76C1a* and *VvCYP86A1a* transcripts showed high expression levels at SGB and RB in comparison with the other stages. Five *VvCYPs* (*VvCYP82C2b*, *VvCYP714A1a*, *VvCYP704A1a*, *VvCYP96A1a*, and *VvCYP711A1b*) were preferentially expressed at high levels in BGB, which indicated that they played a vital role in the fruit development of this stage. *VvCYP89A2a*, *VvCYP72A10a*, and *VvCYP707A1b* showed high expression levels at SGB and BGB in comparison with the other stages and exhibited a varying trend as they increased from SGB to BGB and decreased sharply from BGB to VB then increased progressively toward fruit maturity. *VvCYP84A1a* transcript showed high expression levels at SGB, BGB, and VB and then exhibited a markedly decreased trend toward fruit maturity.

### Transcript Analysis of *VvCYP* Genes in Response to ABA Treatment

**Figure 10:**
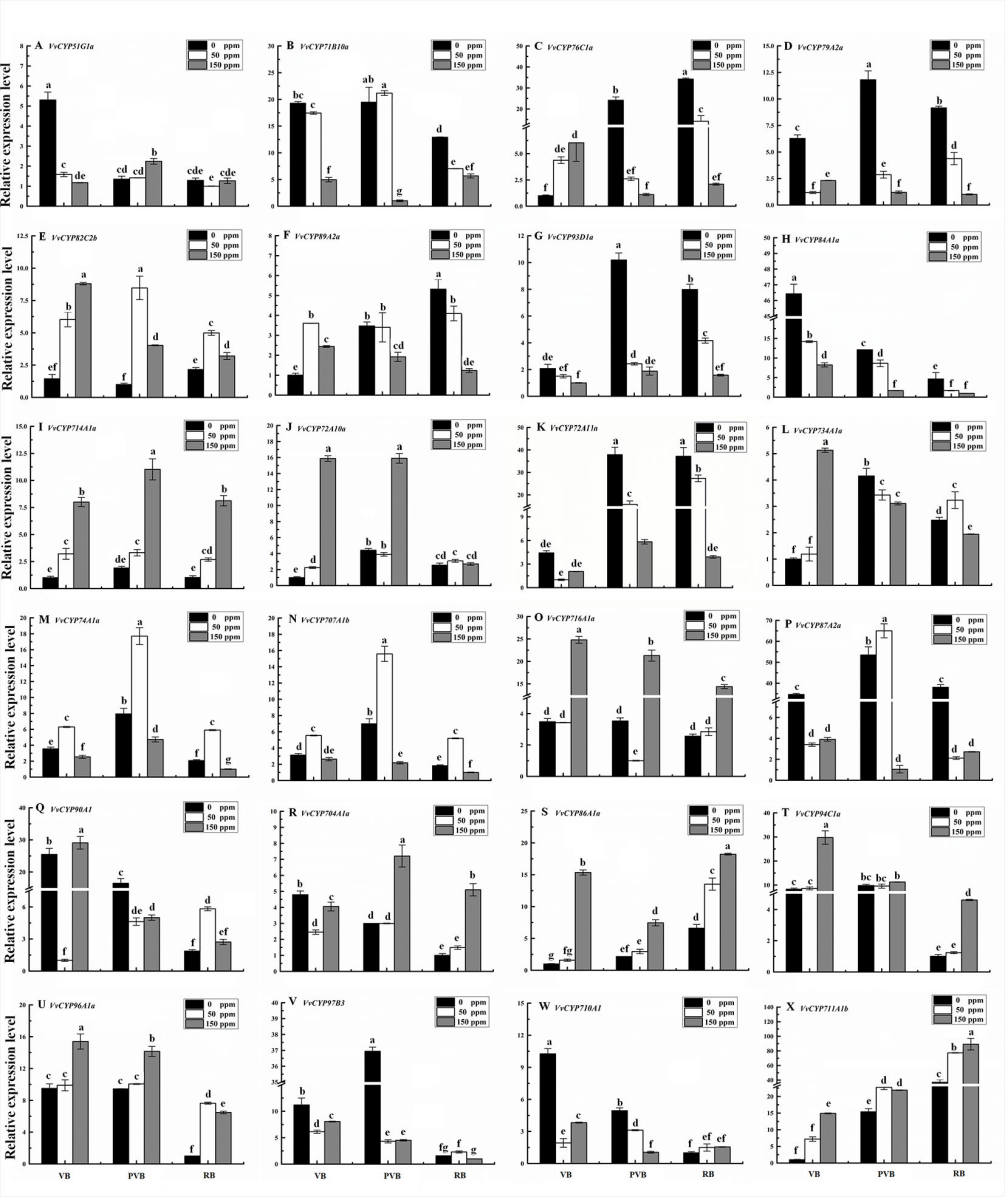
Relative expression levels of *VvCYPs* under ABA treatment. Values were normalized against the expression data of *KyActin1* and are given as means ± standard deviation among three biological replicates. Different letters indicate significant differences between genes (*p*< 0.05). The expression levels were calculated based on the 2 ^−△△Ct^ method. The expression levels of VvCYP51G1a **(A)**, VvCYP71B10a **(B)**, VvCYP76C1a **(C)**, VvCYP79A2a **(D)**, VvCYP82C2b **(E)**, VvCYP89A2a **(F)**, VvCYP93D1a **(G)**, VvCYP84A1a **(H)**, VvCYP714A1a **(I)**, VvCYP72A10a **(J)**, VvCYP72A11a **(K)**, VvCYP734A1a **(L)**, VvCYP74A1a **(M)**, VvCYP707A1b **(N)**, VvCYP716A1a **(O)**, VvCYP87A2a **(P)**, VvCYP90A1 **(Q)**, VvCYP704A1a **(R)**, VvCYP86A1a **(S)**, VvCYP94C1a **(T)**, VvCYP96A1a **(U)**, VvCYP97B3 **(V)**, VvCYP710A1 **(W)**, and VvCYP711A1b **(X)** under ABA treatment.

Abscisic acid (ABA) have engrained roles in plant developmental processes as well as stress signaling networks (Bari and Jones, 2009). As shown in **Table S4**, ABA-responsive *cis*-element, ABRE, was observed in some *VvCYPs*. In order to comprehend how *VvCYPs* express in response to ABA treatment, qRT-PCR was employed to analyze how *VvCYP* transcripts respond to ABA, which has three concentration levels (0 ppm, 50 ppm, and 150 ppm). The expression levels of four *VvCYPs* (*VvCYP82C2b*, *VvCYP714A1a*, *VvCYP86A1a*, and *VvCYP94C1a*) were all enhanced with 50 ppm and 150 ppm ABA compared with 0 ppm ABA at VB, PVB, and RB periods, which is consistent with presence of ABA-responsive *cis*-element (ABRE) in the promoter of these *VvCYPs*, further implying ABA may play an important role in the regulation of grape berry ripening (**Figure 10**). On the contrary, the expression levels of four *VvCYPs* (*VvCYP79A2a*, *VvCYP93D1a*, *VvCYP84A1a*, and *VvCYP72A11a*) were all suppressed with 50 ppm and 150 ppm ABA compared with 0 ppm ABA at VB, PVB, and RB periods. The expression levels of two *VvCYPs* (*VvCYP76C1a* and *VvCYP89A2a*) were all enhanced with 50 ppm and 150 ppm ABA compared with 0 ppm ABA at VB period, whereas they were suppressed with 50 ppm and 150 ppm ABA compared with 0 ppm ABA at PVB and RB periods. The transcript accumulation of two *VvCYPs* (*VvCYP74A1a* and *VvCYP707A1b*) were strongly decreased with 50 ppm ABA compared with 0 ppm ABA at three periods, whereas they were suppressed with 150 ppm ABA compared with 0 ppm ABA at three periods. The expression level of *VvCYP716A1a* was significantly increased with 150 ppm ABA compared with 0 ppm ABA at three periods, and it was not significantly changed with 50 ppm ABA compared with 0 ppm ABA at VB and RB periods; however, it was markedly suppressed with 50 ppm ABA at PVB period. *VvCYP704A1a* showed a significant increase with 150 ppm ABA compared with 0 ppm and 50 ppm ABA at PVB and RB periods.

### Subcellular Localizations

**Figure 11:**
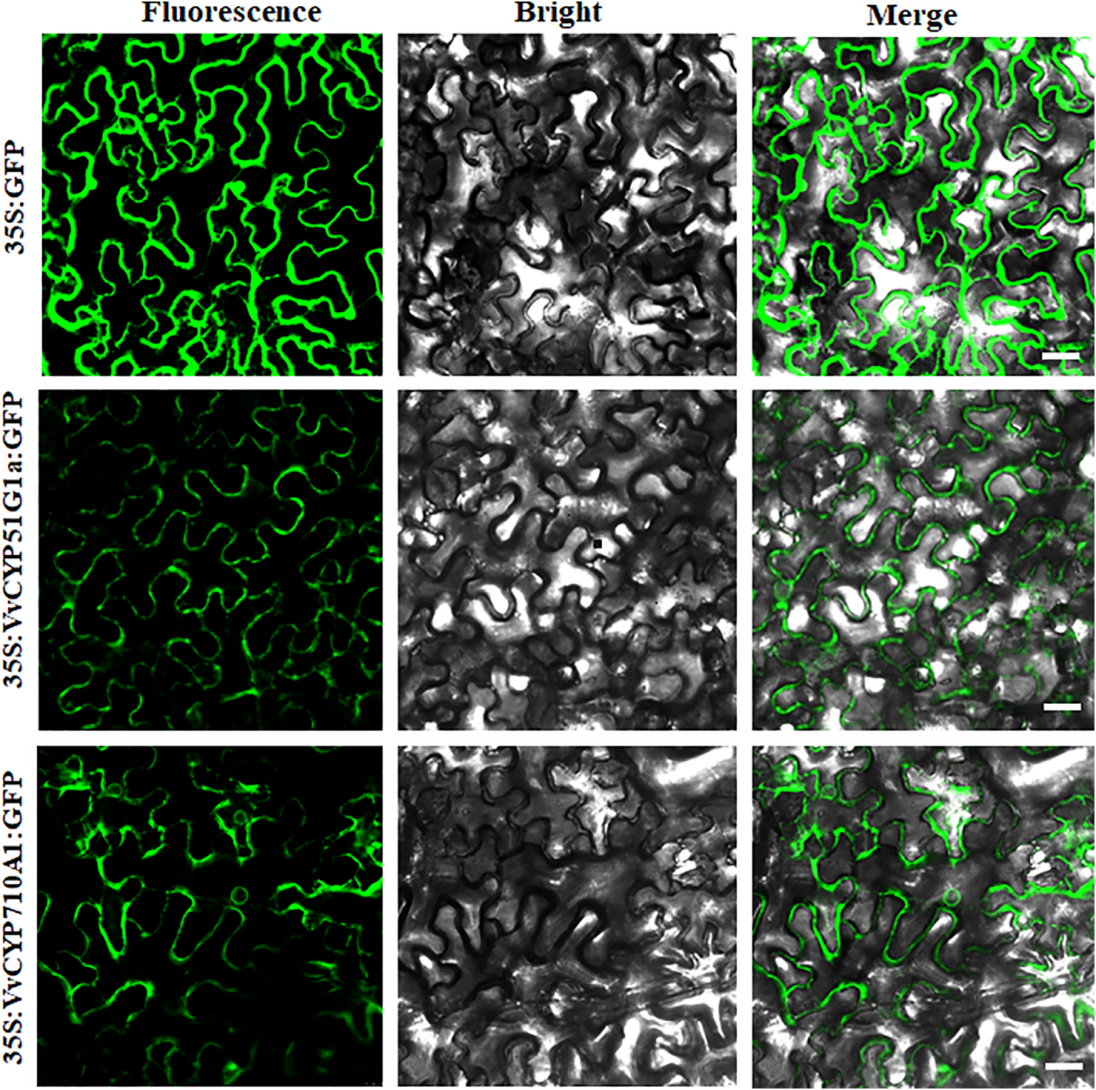
The leaves of 3 to 5-week-old *Nicotiana benthamiana* plants were transiently transformed with control, 35S-VvCYP710A1-GFP, and 35S-VvCYP51G1a-GFP. Images under the blight field (middle), fluorescence (left) and the merged images are shown on the right. Bar: 30 μm.

The subcellular localization of proteins is desirable to explore their biological functions. To investigate the function of VvCYPs, their subcellular localizations were authenticated by the fluorescent protein-tagging method. Firstly, the full-length open reading frames (ORFs) lacking the stop codon of two *VvCYPs* were merged to the N-terminal of the green fluorescence protein (GFP) driven by CaMV 35S promoter, generating fusion proteins p35S-VvCYP51G1a-GFP and p35S-VvCYP710A1-GFP which were agroinfiltrated into leaves of 3 to 5-week-old *N. benthamiana* plants. Fluorescence microscopy exhibited that the PHB-GFP was equally distributed throughout the whole cell, and the fusion proteins of p35S-VvCYP710A1-GFP and p35S-VvCYP51G1a-GFP were both expressed throughout the karyotheca and cytomembrane (**Figure 11**). The results demonstrated that VvCYP710A1 and VvCYP51G1a were both karyotheca- and cytomembrane-localized proteins, suggesting their functional similarity.

## Discussion

The cytochrome P450 (CYP) monooxygenase superfamily, belonging to heme-thiolate protein products, plays a vital role in metabolizing physiologically important compounds in plants (Werck-Reichhart & Feyereisen, 2000). To date, many CYP genes have been reported in some studies with genome-wide characterization, such as, *A. thaliana* (Paquette et al., 2000), *O. sativa* (Wei and Chen, 2018), *P. trichocarpa* (Nelson et al., 2008), and *M. notabilis* (Ma et al., 2014). Nevertheless, no comprehensive analyses of CYP TFs in *V. vinifera* have been done. The present study is the first to report on the genome-wide identification of *VvCYPs* in grapevine, together with evolutionary relations, exon–intron organizations, conservative motifs, *cis*-regulatory elements, gene duplications, and syntenic analysis. Additionally, RNA-seq databases of numerous grapevine tissues were used to determine the expression pattern of all *VvCYPs*, and the selected pivotal genes affecting fruit development and maturity were further verified by qRT-PCR assay.

### CYP Superfamily in *Vitis vinifera* and Their Evolution

In the present study, a sum of 236 *VvCYPs* were identified from the grapevine genome assigned into 46 families, and the number of *VvCYP* members was less than those of *Arabidopsis* (245), *O. sativa* (326), and *P. trichocarpa* (310), and larger than in *M. notabilis* (174), and *C. papaya* (142) (Nelson et al., 2004; Nelson et al., 2008). In contrast, 245 *AtCYPs* in *A. thaliana* were classified into 47 families, of which only *CYP718* was not identified in grapevine. The *OsCYPs* in *O. sativa* were classified into 45 families, of which seven (*CYP729*, *CYP733*, *CYP727*, *CYP728*, *CYP92*, *CYP723*, and *CYP99*) were not found in grapevine. Eight families (*CYP708*, *CYP702*, *CYP720*, *CYP716*, *CYP712*, *CYP705*, *CYP83*, and *CYP82*) were not present in *O*. *sativa*. The quantities of CYP genes in *Arabidopsis* (245) and *O. sativa* (326) were almost 1.04 and 1.38 times more than that in *V. vinifera*, which were inconsistent with the coding genes in the *Arabidopsis* genome (35,386 genes, 135 Mb) and *O. sativa* genome (52,424 genes, 372 Mb) which were about 1.34 and 1.99 times more than that in grapevine (26,346 genes, 487 Mb) (Wang et al., 2012). It was observed that there were more segmental/WGD-type *CYP* genes in *Arabidopsis* and *O. sativa*, suggesting that the enlargement of *CYP* genes in *Arabidopsis* and *O. sativa* might be due to genome duplication events. Therefore, we consider that the difference of genome duplication events may be the main reason for this inconsistency. As shown in **Figure 1**, *VvCYPs* were clustered into nine clans with different functions. *VvCYPs,* which fell into the same clan, probably diverged from a communal ancestor.

Clan 74 which depended on neither NADPH nor molecular oxygen (O_2_) to catalyze interior oxygen transference have possibly arisen in early photosynthetic organisms when O_2_ pressure was still low on the Earth (Stumpe and Feussner, 2006). Clan 51 and clan 85 were clustered together on the phylogenetic tree, the sector may have evolved from a sterol-metabolizing CYP51 ancestor. The members of clan 85 may participate in terpenoid-related pathway, ABA catabolism, and BR biosynthesis (Vriet et al., 2013). We deduced that three clans (clan 72, clan 97, and clan 86) clustering together on the phylogenetic tree may share a common ancestor. Clan 72 is associated with cytokinin (CTK) biosynthesis and the metabolisms of isoprenoid, fatty acid, and phytohormone (GA and BR) (Takei et al., 2004; Kandel et al., 2005; Sakamoto et al., 2011; Magome et al., 2013). Some members of Clan 86 can epoxidate and hydroxylate fatty acid, fatty alcohol, or alkane and their derivatives (Li et al., 2010; Heitz et al., 2012). Clan 711 and clan 71 may have a common origin, and CYP711s play a critical role in the strigolactones (SLs) synthesis (Zhang et al., 2014). The largest clan 71, which consisted of 19 families, indicates a huge functional diversity; it participated in the metabolisms of small isoprenoid and some triterpenoid derivatives, aliphatic and aromatic amino acid derivative, fatty acid, alkaloid and hormone (Sauveplane et al., 2009; Giddings et al., 2011; Wang et al., 2012). The above results provided some powerful evidences that the duplicates diverge to obtain novel functions.

### Role of *Cis*-Elements in the Transcriptional Regulation of *VvCYP* Genes

As the key unit of transcriptional regulation, *cis*-elements participated in the regulation of molecular networks in many biological processes (Ibraheem et al., 2010). In terms of *cis*-regulatory element sequences, the promoters of *VvCYPs* have vastly repetitive regions and some common motifs. In this study, at least 33 *cis*-regulatory elements (ACE, Box 4, Sp1, G-Box, MRE, etc.) were found in the promoter of *VvCYPs* which are required for light-driven transcriptional regulation, implying that these genes played an important role in the light-responsive process. Additionally, the occurrence of the *cis*-element (circadian) indicated the significance of circadian regulation in *VvCYP* gene expression. The results of *cis*-elements analyses showed the presence of unique *cis*-regulatory elements in the promoter of only one *VvCYP* gene, hence deducing the specificity of these gene expression. The unique *cis*-regulatory elements were recognized due to the length of their sequences (6–15 bases) but not usually easy to generate some nucleotide variabilities. Some *VvCYPs* (*e.g. VvCYP82C2b*, *VvCYP714A1a*, *VvCYP86A1a*, and *VvCYP94C1a*) existed in ABA-responsive element (ABRE), which were in accord with the increased expression level of these *VvCYPs* under the ABA treatment. In grapevine, the endosperm plays vital roles in fruit setting as well as the early stage of berry development. Promoter analysis showed that 19 of the 236 *VvCYPs* that harbored GCN4_motif *cis*-elements participated in endosperm expression. Hence, it is concluded that these *VvCYPs* are responsible for fruit development in grapevine.

### The *VvCYP* Superfamily Arose Mainly Through Tandem Duplication, Accompanied by Singleton Duplication

Gene duplication is well known to be the initial dynamic force of new functions in the phylogeny of genomes and genetic systems, which is one of the main evolutionary mechanisms further leading to the divergence and speciation (Lynch, 2000; Moore and Purugganan, 2003). In the current investigations of the grapevine genome, 236 *VvCYPs* were further categorized into 46 families and clustered into 9 clans, mapping in 19 chromosomes or some scaffolds (**Figures 1** and **3**). Based on gene duplication analysis, 114 of all *VvCYPs* in grapevine were duplicated from tandem event, compared with 47 from the singleton event, and 38 from the proximal event, 26 from the dispersed event, and 11 from the WGD/segmental event. The results indicated that during the evolutionary process, some *VvCYPs* have increased rapidly, in which tandem duplication is the major mechanism for *VvCYP* expansion, followed by singleton duplication. However, a diverse phenomenon has been found in the *OsCYP* family of rice (Yin et al., 2013), where majority of the *OsCYP* genes arose through segmental duplication on corresponding chromosomal regions in genetic linkage maps, which is consistent with our findings in grapevine, indicating that the difference of expansion mechanism in grapevine and rice.

### Potential Roles of *VvCYP* Genes in *Vitis vinifera*

Many evidences have already suggested that CYP genes are involved in various biochemical pathways and play multiple roles in the process of growth and development in plants (Werck-Reichhart and Feyereisen, 2000). We attempt to investigate the functions of *VvCYPs* in specific organ and development stage through expression analysis. Some *VvCYPs* were highly expressed in particular flower organs, such as *VvCYP81D5*, *VvCYP89A2i*, *VvCYP94D1*, *VvCYP84A1c*, *VvCYP74B2*, and *VvCYP73A5a* in stamen, *VvCYP707A1b*, *VvCYP716A1b*, *VvCYP82C4d*, *VvCYP89A2a*, and *VvCYP89A2b* in carpel, *VvCYP96A1c*, *VvCYP72A1s*, *VvCYP71A25*, *VvCYP74B2*, and *VvCYP77A4* in petal, *VvCYP89A2a*, *VvCYP82C2k*, *VvCYP82C2d*, *VvCYP94D1*, and *VvCYP71B10e* in pollen (**Figure 7**). Multiple pieces of evidence suggested that *AtCYP704B1*, *AtCYP86C3*, and *AtCYP703A2* catalyzed the hydroxylation of mid- and long-chain fatty acids during pollen exine formation (Xu et al., 2014; Yang et al., 2014; Shi et al., 2015). *VvCYP704A2* shares 67.30% nucleotide sequence identity with *AtCYP704B1*, suggesting a close evolutionary and functional relationship between *VvCYP704A2* and *AtCYP704B1*. *VvCYP703A2* has 69.13% sequence identity with *AtCYP703A2*, indicating a close functional relationship that functions as the hydroxylation of fatty acid (Yang et al., 2014). *VvCYP86A1a* has 69.36% sequence identity with *AtCYP86A8* that functions as a fatty acid *ω*-hydroxylase involved in cutin biosynthesis (Wellesen et al., 2001). Additionally, many *VvCYP* genes were highly expressed in vegetative organs, such as *VvCYP51G1a*, *VvCYP81D2b*, *VvCYP89A2b*, *VvCYP76C1d*, and *VvCYP73A5a* in roots, *VvCYP51G1a*, *VvCYP74A1d*, *VvCYP74B2*, *VvCYP73A5a* and *VvCYP98A3* in stems, *VvCYP71A25* and *VvCYP74B2* in leaves, *VvCYP51G1a*, *VvCYP77A4* and *VvCYP73A5a* in buds.

Leaf development and morphogenesis are regulated by phytohormones, transcriptional regulators, and mechanical properties of the tissue. To explore the VvCYP roles in the processes, qRT-PCR was used to quantify the expression levels along with the different developmental stages (young, medium, and old leaves). The transcripts of seven *VvCYPs* (*VvCYP79A2a*, *VvCYP72A10a*, *VvCYP90A1*, *VvCYP86A1a*, *VvCYP97B3*, *VvCYP710A1* and *VvCYP711A1b*) were down-regulated toward leaf senescence, which suggested that they might function mainly in the upper period of leaf development. *VvCYP87A2c* has 51.96% sequence identity with *OsCYP87A6* that was up-regulated in response to light and auxin signaling and in turn decreased local auxin accumulation in coleoptiles (Chaban et al., 2003). *VvCYP708A2*, a homolog of *AtCYP708A1* which functions upstream of *AtCYP705A5* and downstream of *AtOSC* in the biosynthesis of oxygenated triterpenoids (Field and Osbourn, 2008). *VvCYP94C1a*, a homolog of *AtCYP94C1* that encodes a carotenoid *ϵ*-ring hydroxylase catalyzing the formation of lutein which modulates light energy and serves as a nonphotochemical quenching agent to deal with triplet chlorophyll (Tian et al., 2004; Ding et al., 2017), was expressed at relatively high levels. *VvCYP711A1b* is a homolog of *OsCYP711A5* which may catalyze the biosynthesis of SLs which are carotenoid-derived plant hormones and accelerate leaves' maturity and senescence (Ueda and Kusaba, 2015). Among them, *VvCYP73A5a* has 74.31% sequence identity with *AtCYP73A5* which catalyzes the hydroxylation of t-cinnamic acid, a key early step in the pathway (Pan et al., 2009). Additionally, *VvCYP89A2i* is homologous with *AtCYP89A9* which is implicated in the formation of major chlorophyll catabolites during leaf senescence (Christ et al., 2013). Previous reports suggested that ABA was associated with the regulation of nonclimacteric fruit ripening (Coombe, 1992; Giovannoni, 2001; Li et al., 2011). In the present study, some *VvCYP* transcripts (*VvCYP82C2b*, *VvCYP714A1a*, *VvCYP86A1a*, *VvCYP94C1a*, *VvCYP96A1a*, and *VvCYP711A1b*) were increased with ABA treatment; however, *VvCYP79A2a*, *VvCYP93D1a*, *VvCYP84A1a*, and *VvCYP72A11a* had down-regulated expression after ABA treatment at different berry development stages, implying they might play distinct roles in berry development in response to ABA.

## Data Availability Statement

All relevant data is contained within the article.

## Author Contributions

SJ carried out the experiments, prepared the figures and wrote the manuscript. YX analyzed the data and prepared figures. JW, MA, and LW participated in the collinearity analysis and data analysis. WS and XL contributed to sample collection. IS, CM, and WX contributed with the consultation. CZ managed and designed the research and experiments. CZ and SW revised the manuscript. All authors read and approved the final manuscript.

## Funding

The study was supported by grants from National Postdoctoral Program for Innovative Talents (Grant No. BX20180199), the National Key Research and Development Plan project (Grant No. 2018YFD0201305), China Postdoctoral Science Foundation (Grant No. 2018M642028).

## Conflict of Interest

The authors declare that the research was conducted in the absence of any commercial or financial relationships that could be construed as a potential conﬂict of interest.
