# Supplementary material for: The Cytochrome P450 Monooxygenase Inventory of Grapevine (Vitis vinifera L.): Genome-Wide Identification, Evolutionary Characterization and Expression Analysis
Source: Front Genet. 2020 Feb 18;11:44. doi: 10.3389/fgene.2020.00044 (PMC7040366; doi:10.3389/fgene.2020.00044)
Supplement: Supplementary file 1 [file Table_1.doc]

**Supplementary Table S1.** The primers used for the *VvCYPs* in the present study.

| **Primer Names** | **Sequence (5’-3’)^1^** | **Description^2^** |
| --- | --- | --- |
| VvCYP51G1a-qF1 | TGCATCAAGGAAGCCCTGAG | qRT-PCR |
| VvCYP51G1a-qR1 | AATGCCCCTGCTACCTTGTC | qRT-PCR |
| VvCYP71B10a-qF1 | CATCCGCAGAAGCTGCAAAA | qRT-PCR |
| VvCYP71B10a-qR1 | CTGCACCCTATTCGTGCTGA | qRT-PCR |
| VvCYP76C1a-qF1 | TGTCTTCTTGTCGCCTGGAC | qRT-PCR |
| VvCYP76C1a-qR1 | GGCAAGGTTAGCCAGGGATT | qRT-PCR |
| VvCYP79A2a-qF1 | AAGCCAGTTTTCCGGTGGAT | qRT-PCR |
| VvCYP79A2a-qR1 | TCTTCTTCCACTGCGTTCCC | qRT-PCR |
| VvCYP82C2b-qF1 | GGCCTATCATCGGTCACCTG | qRT-PCR |
| VvCYP82C2b-qR1 | GCTTGGTCGTGTGGAGAAGA | qRT-PCR |
| VvCYP89A2a-qF1 | AGTGGATCATGGCGAACCTG | qRT-PCR |
| VvCYP89A2a-qR1 | TGCATGCGGTACCAGAAAGT | qRT-PCR |
| VvCYP93D1a-qF1 | GACTGCACCATTGGGGGTTA | qRT-PCR |
| VvCYP93D1a-qR1 | TCCACTTCCAAATGGCAGCA | qRT-PCR |
| VvCYP84A1a-qF1 | CGAGTCCGATCTGGAGAAGC | qRT-PCR |
| VvCYP84A1a-qR1 | GGCGGTCTTGTCTCGGTTTA | qRT-PCR |
| VvCYP714A1a-qF1 | AGAGTTCGGTCCTTTGCTCG | qRT-PCR |
| VvCYP714A1a-qR1 | CCCTGCAAGTGATCCGCATA | qRT-PCR |
| VvCYP72A10a-qF1 | TGTTTTCTCGGCGCTTGTTC | qRT-PCR |
| VvCYP72A10a-qR1 | CCACCAGACGCTGTAAACGA | qRT-PCR |
| VvCYP72A11a-qF1 | TGGGTTGGTCCACAAGGAAC | qRT-PCR |
| VvCYP72A11a-qR1 | TGGACCTGAATCCAGGCAAG | qRT-PCR |
| VvCYP734A1a-qF1 | CCCCATCTTAGCCGTCCATC | qRT-PCR |
| VvCYP734A1a-qR1 | GTGCATGTTGGTAGGTGGGA | qRT-PCR |
| VvCYP74A1a-qF1 | CTCGACCCTTCCGAAACCAA | qRT-PCR |
| VvCYP74A1a-qR1 | TTGAAGTCTGCCTCGCCTTT | qRT-PCR |
| VvCYP707A1b-qF1 | CTGGTGACTCGGGCTCATTT | qRT-PCR |
| VvCYP707A1b-qR1 | AGGTGTTAATGACCTGCCCG | qRT-PCR |
| VvCYP716A1a-qF1 | GGACGTGATAGCTCAGAGGC | qRT-PCR |
| VvCYP716A1a-qR1 | GGTAGGGCTATGATTCCGGC | qRT-PCR |
| VvCYP87A2a-qF1 | AATGCTTATGCCTGCGCTTG | qRT-PCR |
| VvCYP87A2a-qR1 | TGGGAGGATCGATTTTGGGC | qRT-PCR |
| VvCYP90A1-qF1 | AAGGCAAGGACGAAGGTAGC | qRT-PCR |
| VvCYP90A1-qR1 | ATGGTGGAGGTGGTCTCGTA | qRT-PCR |
| VvCYP704A1a-qF1 | GGCCCGAGAATTTGTTTGGG | qRT-PCR |
| VvCYP704A1a-qR1 | GAAAGCACGAACATGGAGGC | qRT-PCR |
| VvCYP86A1a-qF1 | CTCTGAAGGGTCCACGTGTC | qRT-PCR |
| VvCYP86A1a-qR1 | CGAGCAGGTCATGGAACACT | qRT-PCR |
| VvCYP94C1a-qF1 | GCTTCGAACAAATGCGGGAG | qRT-PCR |
| VvCYP94C1a-qR1 | ATGCTTTCTCCATACGCCCC | qRT-PCR |
| VvCYP96A1a-qF1 | CAGATGTCCTACCGAGTGGC | qRT-PCR |
| VvCYP96A1a-qR1 | GCTACCCCCTTCCCTAGACA | qRT-PCR |
| VvCYP97B3-qF1 | ATAACCTCCATCGGTCCCCA | qRT-PCR |
| VvCYP97B3-qR1 | CCTCATTGGGGTACAACGCT | qRT-PCR |
| VvCYP710A1-qF1 | GTTCCCATCGGTGTTCGAGT | qRT-PCR |
| VvCYP710A1-qR1 | CCATCCGTTCTGTGCCTCTT | qRT-PCR |
| VvCYP711A1b-qF1 | TGCCAACCATGCAAGCATTC | qRT-PCR |
| VvCYP711A1b-qR1 | GCTGGATGGTGGTTTGGAGA | qRT-PCR |
| Actin-qF | GATTCTGGTGATGGTGTGAGT | qRT-PCR |
| Actin -qR | GACAATTTCCCGTTCAGCAGT | qRT-PCR |
| VvCYP51G1a-F1 | ACCAGTCTCTCTCTCAAGCTTATGGATGTAGACAACAAGTTC | Subcellular localization |
| VvCYP51G1a-R1 | GCTCACCATGGATCCAAGCTTGTCAACAGGAAGTACTCGGCG | Subcellular localization |
| VvCYP710A1-F1 | ACCAGTCTCTCTCTCAAGCTTATGGAGTCTTCTTCTACGTTG | Subcellular localization |
| VvCYP710A1-R1 | GCTCACCATGGATCCAAGCTTAGAGAAGGAGGGATAGCGTGC | Subcellular localization |

1 Restriction sites are indicated in red.

2 The type of experiment for which the primers were used is indicated in brackets (qRT-PCR: quantitative reverse transcription PCR).
