# Supplementary material for: The Cytochrome P450 Monooxygenase Inventory of Grapevine (Vitis vinifera L.): Genome-Wide Identification, Evolutionary Characterization and Expression Analysis
Source: Front Genet. 2020 Feb 18;11:44. doi: 10.3389/fgene.2020.00044 (PMC7040366; doi:10.3389/fgene.2020.00044)
Supplement: Supplementary file 2 [file Table_2.docx]

| **Table S2.** Structural and biochemical information of identified VvCYP members in grapevine. | | | | | | | | | | | | | |
| --- | --- | --- | --- | --- | --- | --- | --- | --- | --- | --- | --- | --- | --- |
| No. | Gene name | Gene ID | Chromosome Location | Strand | ORF  (bp) | Deduced polypeptide | | | TMD^c^ | Subcellular localization | Gene duplication | Family | Clan |
|  |  |  |  |  |  | Length (aa) | MW  (kDa) | pI |  |  |  |  |  |
| 1 | VvCYP51G1a | VIT_201s0150g00210.1 | chr1:22640127..22644443 | reverse | 1461 | 486 | 121.15 | 4.98 | 1 | E.R.:4.5,E.R._plas:3.5,cyto:3,extr:3,mito:2,plas:1.5 | Tandem | CYP51 | 51 |
| 2 | VvCYP51G1b | VIT_213s0047g01200.1 | chr13:18253613..18264996 | forward | 1461 | 486 | 121.39 | 5.00 | 1 | E.R.:4.5,E.R._plas:3.5,cyto:3,mito:2,extr:2,plas:1.5,chlo:1 | Tandem |  |  |
| 3 | VvCYP701A3 | VIT_218s0001g11320.1 | chr18:9624043..9629089 | reverse | 1527 | 508 | 124.32 | 5.02 | 1 | mito:6,nucl:5,cyto:2,chlo:1 | Singleton | CYP701 | 71 |
| 4 | VvCYP703A2 | VIT_215s0046g00330.1 | chr15:17343704..17345642 | forward | 1575 | 524 | 129.69 | 4.96 | 1 | plas:7,vacu:4,cyto:1,extr:1,golg:1 | Singleton | CYP703 |  |
| 5 | VvCYP705A2 | VIT_213s0064g01320.1 | chr13:23193677..23196827 | reverse | 1548 | 515 | 127.48 | 4.99 | 1 | plas:9,golg:2,cyto:1,extr:1,vacu:1 | Singleton | CYP705 |  |
| 6 | VvCYP705A3a | VIT_210s0092g00480.1 | chr10:12042867..12045200 | forward | 1518 | 505 | 126.08 | 5.01 | 0 | nucl:6,mito:5,cyto:2,extr:1 | Tandem |  |  |
| 7 | VvCYP705A3b | VIT_210s0092g00510.1 | chr10:12078341..12080144 | forward | 1344 | 447 | 111.36 | 5.04 | 0 | nucl:6,mito:5,cyto:2,extr:1 | Tandem |  |  |
| 8 | VvCYP71B10a | VIT_201s0137g00510.1 | chr1:7005096..7007581 | reverse | 1512 | 503 | 125.21 | 5.00 | 0 | nucl:6,mito:5,cyto:2,extr:1 | Proximal | CYP71 |  |
| 9 | VvCYP71B10b | VIT_201s0137g00520.1 | chr1:7022158..7024645 | reverse | 1518 | 505 | 125.30 | 5.00 | 1 | nucl:6,mito:5,cyto:2,extr:1 | Proximal |  |  |
| 10 | VvCYP71B10c | VIT_201s0137g00540.1 | chr1:7027620..7030338 | reverse | 1518 | 505 | 125.66 | 4.99 | 1 | E.R.:5.5,E.R._plas:4,extr:3,cyto:2,mito:2,plas:1.5 | Proximal |  |  |
| 11 | VvCYP71B10d | VIT_201s0137g00550.1 | chr1:7040351..7042753 | reverse | 1518 | 505 | 125.35 | 5.00 | 0 | nucl:6,mito:5,cyto:2,extr:1 | Tandem |  |  |
| 12 | VvCYP71B10e | VIT_201s0137g00560.1 | chr1:7047621..7050242 | reverse | 1518 | 505 | 125.56 | 4.99 | 0 | nucl:6,mito:5,cyto:2,extr:1 | Tandem |  |  |
| 13 | VvCYP71B24a | VIT_206s0004g06480.1 | chr6:7204985..7207180 | forward | 1575 | 524 | 130.98 | 4.98 | 1 | plas:8,vacu:3,cyto:1,extr:1,golg:1 | Singleton |  |  |
| 14 | VvCYP71A14a | VIT_217s0000g09510.1 | chr17:11179330..11181788 | reverse | 1548 | 515 | 127.32 | 5.01 | 1 | vacu:7,plas:4,extr:2,cyto:1 | Singleton |  |  |
| 15 | VvCYP71A25 | VIT_217s0000g09550.1 | chr17:11202641..11204433 | forward | 876 | 291 | 71.09 | 5.15 | 1 | nucl:6,mito:5,cyto:2,extr:1 | Tandem |  |  |
| 16 | VvCYP71A22 | VIT_217s0000g09570.1 | chr17:11284591..11286979 | forward | 1542 | 513 | 127.28 | 4.98 | 1 | E.R.:4.5,cyto:3.5,E.R._plas:3.5,cyto_pero:2.5,mito:2,plas:1.5,chlo:1,extr:1 | Tandem |  |  |
| 17 | VvCYP71B4 | VIT_219s0015g00100.1 | chr19:8064803..8067438 | reverse | 1527 | 508 | 126.49 | 5.00 | 0 | E.R.:5.5,E.R._plas:4,extr:3,cyto:2,mito:2,plas:1.5 | Tandem |  |  |
| 18 | VvCYP71B24b | VIT_218s0001g13820.1 | chr18:11794593..11796161 | reverse | 1497 | 498 | 123.79 | 5.00 | 1 | cyto:4.5,E.R.:3.5,cyto_pero:3,E.R._plas:3,mito:2,plas:1.5,chlo:1,extr:1 | Tandem |  |  |
| 19 | VvCYP71B24c | VIT_218s0001g13830.1 | chr18:11801810..11803547 | reverse | 1497 | 498 | 123.92 | 5.00 | 1 | cyto:4.5,E.R.:3.5,cyto_pero:3,E.R._plas:3,mito:2,plas:1.5,chlo:1,extr:1 | Tandem |  |  |
| 20 | VvCYP71B3a | VIT_218s0001g13850.1 | chr18:11832255..11834105 | reverse | 1491 | 496 | 123.12 | 5.01 | 1 | cyto:4.5,E.R.:3.5,cyto_pero:3,E.R._plas:3,mito:2,plas:1.5,chlo:1,extr:1 | Tandem |  |  |
| 21 | VvCYP71B23 | VIT_219s0015g00080.1 | chr19:8052492..8054394 | reverse | 1530 | 509 | 127.23 | 4.99 | 0 | E.R.:4.5,E.R._plas:3.5,cyto:3,extr:3,mito:2,plas:1.5 | Dispersed |  |  |
| 22 | VvCYP71A14b | VIT_202s0025g04360.1 | chr2:3833479..3835686 | reverse | 1515 | 504 | 125.86 | 4.99 | 1 | nucl:6,mito:5,cyto:2,extr:1 | Tandem |  |  |
| 23 | VvCYP71A23 | VIT_203s0063g01480.1 | chr3:4928346..4930604 | forward | 1617 | 538 | 133.16 | 4.97 | 1 | nucl:6,mito:5,cyto:2,extr:1 | Proximal |  |  |
| 24 | VvCYP71B10g | VIT_206s0004g06460.1 | chr6:7192185..7194190 | forward | 1596 | 531 | 132.07 | 4.97 | 1 | E.R.:5.5,E.R._plas:4,extr:3,cyto:2,mito:2,plas:1.5 | Proximal |  |  |
| 25 | VvCYP71B31 | VIT_208s0007g04100.1 | chr8:18077622..18080310 | reverse | 1557 | 518 | 126.70 | 4.99 | 1 | plas:8,vacu:3,extr:2,cyto:1 | Proximal |  |  |
| 26 | VvCYP71B3b | VIT_208s0032g00240.1 | chr8:3263007..3265066 | forward | 1515 | 504 | 124.86 | 5.01 | 0 | nucl:5,mito:5,cyto:3,extr:1 | Proximal |  |  |
| 27 | VvCYP71B3c | VIT_210s0092g00370.1 | chr10:11930292..11932586 | forward | 1509 | 502 | 125.75 | 5.01 | 0 | nucl:5,mito:5,cyto:3,extr:1 | Tandem |  |  |
| 28 | VvCYP71B10h | VIT_217s0000g09610.1 | chr17:11349787..11352003 | forward | 1524 | 507 | 126.82 | 4.98 | 1 | nucl:6,mito:5,cyto:2,extr:1 | Singleton |  |  |
| 29 | VvCYP71B20 | VIT_218s0001g13760.1 | chr18:11761760..11763067 | reverse | 954 | 317 | 77.80 | 5.10 | 0 | cyto:4.5,E.R.:3.5,cyto_pero:3,E.R._plas:3,mito:2,plas:1.5,chlo:1,extr:1 | Tandem |  |  |
| 30 | VvCYP71B10i | VIT_218s0001g13770.1 | chr18:11767436..11769613 | reverse | 1491 | 496 | 123.73 | 5.00 | 0 | nucl:5,mito:5,cyto:3,extr:1 | Tandem |  |  |
| 31 | VvCYP71B10j | VIT_218s0001g13780.1 | chr18:11774186..11776264 | reverse | 1485 | 494 | 123.47 | 5.00 | 1 | mito:6,nucl:5,cyto:2,chlo:1 | Tandem |  |  |
| 32 | VvCYP71B10k | VIT_218s0001g13790.1 | chr18:11779188..11781395 | reverse | 1488 | 495 | 123.50 | 5.00 | 1 | plas:10,chlo:1,cyto:1,extr:1,vacu:1 | Tandem |  |  |
| 33 | VvCYP712A1a | VIT_212s0059g02070.1 | chr12:6864980..6866937 | forward | 1575 | 524 | 129.57 | 4.97 | 1 | E.R.:5.5,E.R._plas:4,extr:3,cyto:2,mito:2,plas:1.5 | Proximal | CYP712 |  |
| 34 | VvCYP73A5a | VIT_206s0004g08150.1 | chr6:8881464..8886329 | reverse | 1518 | 505 | 123.46 | 4.99 | 1 | E.R.:4.5,E.R._plas:3.5,cyto:3,extr:3,mito:2,plas:1.5 | Singleton | CYP73 |  |
| 35 | VvCYP73A5b | VIT_211s0065g00350.1 | chr11:14089792..14092260 | reverse | 1599 | 532 | 132.95 | 4.95 | 1 | E.R.:5.5,E.R._plas:4,extr:3,cyto:2,mito:2,plas:1.5 | Dispersed |  |  |
| 36 | VvCYP73A5c | VIT_211s0078g00290.1 | chr11:12503269..12505262 | forward | 1605 | 534 | 133.54 | 4.96 | 2 | E.R.:5.5,E.R._plas:4,extr:3,cyto:2,mito:2,plas:1.5 | Dispersed |  |  |
| 37 | VvCYP75B1a | VIT_217s0000g07200.1 | chr17:8011519..8014480 | forward | 1530 | 509 | 126.41 | 4.93 | 1 | plas:8,vacu:3,cyto:1,extr:1,golg:1 | Tandem | CYP75 |  |
| 38 | VvCYP75B1b | VIT_217s0000g07210.1 | chr17:8036250..8037753 | forward | 1077 | 358 | 87.05 | 5.02 | 0 | plas:10,cyto:1,extr:1,vacu:1,golg:1 | Tandem |  |  |
| 39 | VvCYP76C1a | VIT_202s0012g02340.1 | chr2:9295170..9300215 | forward | 1497 | 498 | 124.16 | 4.98 | 0 | nucl:6,mito:6,chlo:1,cyto:1 | Tandem | CYP76 |  |
| 40 | VvCYP76C1b | VIT_202s0025g04864.1 | chr2:4394176..4401372 | forward | 1500 | 499 | 124.16 | 4.98 | 0 | plas:11,cyto:1,vacu:1,golg:1 | Tandem |  |  |
| 41 | VvCYP76C1d | VIT_202s0025g04880.1 | chr2:4399667..4401305 | forward | 1245 | 414 | 102.26 | 5.03 | 0 | vacu:6,plas:5,extr:2,cyto:1 | Tandem |  |  |
| 42 | VvCYP76C1e | VIT_215s0048g01470.1 | chr15:15595580..15598731 | forward | 1500 | 499 | 126.47 | 4.96 | 1 | plas:7,vacu:4,cyto:1,extr:1,golg:1 | Tandem |  |  |
| 43 | VvCYP76C1f | VIT_215s0048g01490.1 | chr15:15609541..15612332 | forward | 1500 | 499 | 126.22 | 4.96 | 1 | plas:8,vacu:3,cyto:1,extr:1,golg:1 | Tandem |  |  |
| 44 | VvCYP76C1g | VIT_215s0048g01500.1 | chr15:15635769..15636949 | reverse | 834 | 277 | 68.23 | 5.12 | 0 | E.R.:4.5,cyto:3.5,E.R._plas:3.5,cyto_pero:2.5,mito:2,plas:1.5,chlo:1,extr:1 | Tandem |  |  |
| 45 | VvCYP76C1h | VIT_215s0048g01515.1 | chr15:15655036..15656979 | reverse | 1083 | 360 | 89.87 | 5.06 | 0 | nucl:5,mito:5,cyto:3,extr:1 | Tandem |  |  |
| 46 | VvCYP76C1i | VIT_215s0048g01530.1 | chr15:15688558..15694452 | reverse | 1491 | 496 | 124.77 | 4.98 | 0 | nucl:6,mito:5,cyto:2,extr:1 | Tandem |  |  |
| 47 | VvCYP76C1l | VIT_215s0048g01600.1 | chr15:15741593..15744038 | reverse | 1491 | 496 | 124.34 | 4.98 | 0 | E.R.:4.5,E.R._plas:3.5,cyto:3,extr:3,mito:2,plas:1.5 | Tandem |  |  |
| 48 | VvCYP76C4b | VIT_202s0012g02810.1 | chr2:10784811..10786906 | reverse | 1491 | 496 | 126.46 | 4.96 | 1 | nucl:6,mito:5,cyto:2,extr:1 | Tandem |  |  |
| 49 | VvCYP76C4c | VIT_202s0012g02820.1 | chr2:10832753..10835365 | reverse | 1491 | 496 | 126.53 | 4.96 | 1 | nucl:6,mito:5,cyto:2,extr:1 | Tandem |  |  |
| 50 | VvCYP76C4d | VIT_202s0109g00310.1 | chr2:12824085..12826782 | reverse | 1506 | 501 | 127.68 | 4.96 | 1 | plas:7,vacu:4,cyto:1,extr:1,golg:1 | Tandem |  |  |
| 51 | VvCYP76C4e | VIT_203s0097g00460.1 | chr3:10701170..10703581 | forward | 1491 | 496 | 126.37 | 4.96 | 1 | E.R.:4.5,E.R._plas:4,cyto:3,plas:2.5,mito:2,extr:2 | Dispersed |  |  |
| 52 | VvCYP76C5c | VIT_208s0007g04040.1 | chr8:18038023..18039925 | reverse | 1536 | 511 | 125.18 | 4.99 | 0 | cyto:4.5,E.R.:3.5,cyto_pero:3,E.R._plas:3,mito:2,plas:1.5,chlo:1,extr:1 | Singleton |  |  |
| 53 | VvCYP76C2a | VIT_208s0007g04120.1 | chr8:18098799..18101546 | reverse | 1527 | 508 | 124.25 | 4.99 | 1 | plas:11,cyto:1,extr:1,vacu:1 | Proximal |  |  |
| 54 | VvCYP77B1 | VIT_210s0116g00800.1 | chr10:364436..365968 | forward | 1527 | 508 | 124.48 | 4.96 | 0 | E.R.:5.5,E.R._plas:4,extr:3,cyto:2,mito:2,plas:1.5 | Singleton | CYP77 |  |
| 55 | VvCYP77A4 | VIT_213s0074g00390.1 | chr13:8019969..8021714 | reverse | 1548 | 515 | 127.95 | 4.96 | 1 | plas:10,cyto:1,extr:1,vacu:1,golg:1 | Singleton |  |  |
| 56 | VvCYP78A5 | VIT_201s0010g03560.1 | chr1:20910069..20913845 | forward | 1551 | 516 | 128.96 | 4.97 | 1 | E.R.:5.5,E.R._plas:4,extr:3,cyto:2,mito:2,plas:1.5 | Singleton | CYP78 |  |
| 57 | VvCYP78A10a | VIT_201s0011g00350.1 | chr1:380874..383136 | reverse | 1557 | 518 | 127.44 | 4.98 | 1 | E.R.:5.5,E.R._plas:4,extr:3,cyto:2,mito:2,plas:1.5 | Singleton |  |  |
| 58 | VvCYP78A6 | VIT_202s0025g02570.1 | chr2:2231607..2233740 | reverse | 1581 | 526 | 130.78 | 4.94 | 1 | plas:10,cyto:1,extr:1,vacu:1,golg:1 | Singleton |  |  |
| 59 | VvCYP78A7a | VIT_207s0031g01570.1 | chr7:17663976..17665760 | reverse | 1620 | 539 | 134.30 | 4.94 | 2 | plas:8,vacu:3,extr:2,cyto:1 | Singleton |  |  |
| 60 | VvCYP78A10b | VIT_215s0048g02900.1 | chr15:17005314..17007669 | forward | 1602 | 533 | 133.28 | 4.94 | 1 | nucl:6,mito:5,cyto:2,extr:1 | Dispersed |  |  |
| 61 | VvCYP78A10c | VIT_217s0000g05110.1 | chr17:5600225..5602640 | reverse | 1578 | 525 | 129.73 | 5.01 | 2 | nucl:6,mito:5,cyto:2,extr:1 | Dispersed |  |  |
| 62 | VvCYP78A7b | VIT_218s0001g00590.1 | chr18_random:1435268..1437180 | forward | 1623 | 540 | 135.37 | 4.95 | 1 | nucl:6,mito:5,cyto:2,extr:1 | Singleton |  |  |
| 63 | VvCYP79A2a | VIT_206s0009g01780.1 | chr6:13799987..13801712 | reverse | 1635 | 544 | 134.77 | 4.97 | 1 | cyto:4.5,E.R.:3.5,cyto_pero:3,E.R._plas:3,mito:2,plas:1.5,chlo:1,extr:1 | Dispersed | CYP79 |  |
| 64 | VvCYP79A2b | VIT_206s0009g03120.1 | chr6:16266089..16267917 | forward | 1554 | 517 | 128.36 | 4.98 | 1 | E.R.:5.5,E.R._plas:4,extr:3,cyto:2,mito:2,plas:1.5 | Tandem |  |  |
| 65 | VvCYP79A2e | VIT_213s0067g02730.1 | chr13:1467798..1469637 | reverse | 1740 | 579 | 144.19 | 4.95 | 1 | E.R.:5.5,E.R._plas:4,extr:3,cyto:2,mito:2,plas:1.5 | Tandem |  |  |
| 66 | VvCYP79A2f | VIT_213s0067g02780.1 | chr13:1519993..1521580 | reverse | 1347 | 448 | 110.08 | 5.02 | 0 | nucl:6,mito:5,cyto:2,extr:1 | Tandem |  |  |
| 67 | VvCYP79A2g | VIT_213s0067g02790.1 | chr13:1526954..1528642 | reverse | 1107 | 368 | 90.32 | 5.06 | 0 | nucl:6,mito:5,cyto:2,extr:1 | Tandem |  |  |
| 68 | VvCYP79A2h | VIT_213s0067g02830.1 | chr13:1541944..1543728 | reverse | 1521 | 506 | 126.35 | 4.98 | 1 | E.R.:5.5,E.R._plas:4,extr:3,cyto:2,mito:2,plas:1.5 | Tandem |  |  |
| 69 | VvCYP79A2i | VIT_213s0106g00280.1 | chr13:9834087..9835851 | forward | 1653 | 550 | 137.03 | 4.97 | 1 | nucl:6,mito:5,cyto:2,extr:1 | Dispersed |  |  |
| 70 | VvCYP81D1a | VIT_200s0705g00010.1 | chrUn:34480727..34482204 | reverse | 1098 | 365 | 89.08 | 5.09 | 0 | nucl:6,mito:5,cyto:2,extr:1 | Dispersed | CYP81 |  |
| 71 | VvCYP81D1b | VIT_200s0790g00020.1 | chrUn:35411707..35413967 | reverse | 1686 | 561 | 140.40 | 4.95 | 1 | E.R.:5.5,E.R._plas:4,extr:3,cyto:2,mito:2,plas:1.5 | Dispersed |  |  |
| 72 | VvCYP81D1d | VIT_207s0129g00710.1 | chr7:15956381..15958232 | forward | 1509 | 502 | 125.37 | 4.97 | 1 | vacu:6,plas:5,extr:2,cyto:1 | Proximal |  |  |
| 73 | VvCYP81D1e | VIT_207s0129g00730.1 | chr7:15965349..15967409 | reverse | 1521 | 506 | 126.38 | 5.00 | 1 | E.R.:5.5,E.R._plas:4,extr:3,cyto:2,mito:2,plas:1.5 | Tandem |  |  |
| 74 | VvCYP81D1f | VIT_207s0129g00760.1 | chr7:15983253..15985253 | forward | 1515 | 504 | 126.03 | 4.97 | 1 | plas:7,vacu:4,extr:2,cyto:1 | Proximal |  |  |
| 75 | VvCYP81D4b | VIT_207s0129g00800.1 | chr7:16015531..16017755 | forward | 1515 | 504 | 125.73 | 4.97 | 1 | plas:7,vacu:4,extr:2,cyto:1 | Proximal |  |  |
| 76 | VvCYP81D1h | VIT_207s0129g00810.1 | chr7:16023324..16024845 | forward | 1071 | 356 | 87.00 | 5.11 | 0 | nucl:5,mito:5,cyto:3,extr:1 | Tandem |  |  |
| 77 | VvCYP81D1i | VIT_207s0129g00820.1 | chr7:16026832..16029009 | forward | 1497 | 498 | 124.58 | 5.00 | 1 | E.R.:4.5,E.R._plas:3.5,cyto:3,mito:2,extr:2,plas:1.5,chlo:1 | Tandem |  |  |
| 78 | VvCYP81D1j | VIT_207s0129g00830.1 | chr7:16039693..16041669 | forward | 810 | 269 | 65.68 | 5.15 | 0 | nucl:6,mito:5,cyto:2,extr:1 | Proximal |  |  |
| 79 | VvCYP81D2a | VIT_207s0129g00850.1 | chr7:16055528..16057950 | forward | 1500 | 499 | 123.76 | 4.99 | 2 | E.R.:5.5,E.R._plas:4,extr:3,cyto:2,mito:2,plas:1.5 | Proximal |  |  |
| 80 | VvCYP81D3a | VIT_209s0002g06450.1 | chr9:6390724..6392566 | reverse | 1524 | 507 | 124.57 | 4.98 | 0 | plas:8,vacu:3,extr:2,cyto:1 | Tandem |  |  |
| 81 | VvCYP81H1 | VIT_209s0002g06470.1 | chr9:6420581..6422433 | forward | 1539 | 512 | 125.87 | 4.99 | 0 | nucl:6,mito:5,cyto:2,extr:1 | Tandem |  |  |
| 82 | VvCYP81D2b | VIT_214s0030g01940.1 | chr14:6898973..6901562 | reverse | 1497 | 498 | 123.61 | 4.99 | 2 | E.R.:5.5,E.R._plas:4,extr:3,cyto:2,mito:2,plas:1.5 | Dispersed |  |  |
| 83 | VvCYP81D3b | VIT_218s0001g09500.1 | chr18:7942173..7954535 | reverse | 1497 | 498 | 123.43 | 4.98 | 1 | E.R.:4.5,E.R._plas:3.5,cyto:3,extr:3,mito:2,plas:1.5 | Tandem |  |  |
| 84 | VvCYP81D5 | VIT_218s0001g09650.1 | chr18:8045533..8049357 | forward | 1524 | 507 | 126.35 | 4.97 | 1 | plas:8,vacu:3,cyto:1,extr:1,golg:1 | Singleton |  |  |
| 85 | VvCYP81D1l | VIT_218s0001g09660.1 | chr18:8052983..8055170 | forward | 1533 | 510 | 126.78 | 4.98 | 0 | E.R.:4.5,E.R._plas:3.5,cyto:3,extr:3,mito:2,plas:1.5 | Singleton |  |  |
| 86 | VvCYP81D4c | VIT_207s0129g00780.1 | chr7:16006633..16007169 | forward | 1620 | 539 | 134.30 | 4.94 | 2 | plas:8,vacu:3,extr:2,cyto:1 | Proximal |  |  |
| 87 | VvCYP81D2c | VIT_209s0002g06440.1 | chr9:6372932..6374662 | reverse | 1527 | 508 | 124.81 | 4.98 | 0 | E.R.:4.5,E.R._plas:3.5,cyto:3,extr:3,mito:2,plas:1.5 | Tandem |  |  |
| 88 | VvCYP81D8 | VIT_209s0002g06480.1 | chr9:6425996..6427676 | forward | 1416 | 471 | 114.34 | 5.02 | 0 | nucl:5,mito:5,cyto:3,extr:1 | Tandem |  |  |
| 89 | VvCYP81D4d | VIT_218s0001g09520.1 | chr18:7958972..7961800 | reverse | 1659 | 552 | 137.43 | 4.97 | 1 | plas:8,vacu:3,extr:2,cyto:1 | Singleton |  |  |
| 90 | VvCYP82C4a | VIT_203s0063g01630.1 | chr3:5003738..5004868 | forward | 822 | 273 | 67.92 | 5.11 | 0 | mito:6,nucl:5,cyto:2,extr:1 | Tandem | CYP82 |  |
| 91 | VvCYP82C3b | VIT_203s0063g01690.1 | chr3:5037094..5039672 | forward | 1584 | 527 | 130.37 | 4.98 | 2 | E.R.:5.5,E.R._plas:4,extr:3,cyto:2,mito:2,plas:1.5 | Proximal |  |  |
| 92 | VvCYP82C2b | VIT_207s0255g00090.1 | chr7:14414497..14417447 | forward | 1587 | 528 | 130.35 | 4.99 | 2 | E.R.:5.5,E.R._plas:4,extr:3,cyto:2,mito:2,plas:1.5 | Singleton |  |  |
| 93 | VvCYP82C2c | VIT_209s0002g09250.1 | chr9:11038395..11043868 | reverse | 1557 | 518 | 128.58 | 4.98 | 1 | E.R.:4.5,E.R._plas:4,extr:3,plas:2.5,cyto:2,mito:2 | Tandem |  |  |
| 94 | VvCYP82C2d | VIT_209s0002g09270.1 | chr9:11097756..11101686 | reverse | 1011 | 336 | 82.83 | 5.08 | 0 | nucl:6,mito:5,cyto:2,extr:1 | Tandem |  |  |
| 95 | VvCYP82C2e | VIT_212s0035g00570.1 | chr12:19947032..19949576 | reverse | 1455 | 484 | 120.73 | 4.99 | 0 | nucl:6,mito:5,cyto:2,extr:1 | Tandem |  |  |
| 96 | VvCYP82C2f | VIT_218s0001g11400.1 | chr18:9699647..9701942 | forward | 1614 | 537 | 134.05 | 4.96 | 2 | nucl:6,mito:5,cyto:2,extr:1 | Singleton |  |  |
| 97 | VvCYP82C3c | VIT_218s0001g11430.1 | chr18:9725991..9731095 | forward | 1545 | 514 | 127.31 | 5.00 | 0 | plas:8,vacu:3,extr:2,cyto:1 | Proximal |  |  |
| 98 | VvCYP82C4b | VIT_218s0001g11470.1 | chr18:9754020..9758959 | forward | 1545 | 514 | 127.16 | 5.00 | 0 | E.R.:4.5,E.R._plas:4,cyto:3,plas:2.5,mito:2,extr:2 | Proximal |  |  |
| 99 | VvCYP82C4c | VIT_218s0001g11490.1 | chr18:9788462..9791476 | forward | 1578 | 525 | 129.95 | 5.00 | 2 | nucl:6,mito:5,cyto:2,extr:1 | Proximal |  |  |
| 100 | VvCYP82C3d | VIT_218s0001g11500.1 | chr18:9794689..9797337 | forward | 1581 | 526 | 131.09 | 4.98 | 2 | nucl:6,mito:5,cyto:2,extr:1 | Proximal |  |  |
| 101 | VvCYP82C2k | VIT_209s0002g09200.1 | chr9:10966926..10971504 | reverse | 1632 | 543 | 134.55 | 4.97 | 1 | nucl:5,mito:5,cyto:3,extr:1 | Tandem |  |  |
| 102 | VvCYP82C4d | VIT_211s0016g01020.1 | chr11:856210..858446 | forward | 1530 | 509 | 125.56 | 4.99 | 0 | plas:10,golg:2,cyto:1,vacu:1 | Tandem |  |  |
| 103 | VvCYP82C2l | VIT_212s0035g00560.1 | chr12:19929658..19932528 | reverse | 1437 | 478 | 118.95 | 5.00 | 1 | nucl:6,mito:5,cyto:2,extr:1 | Tandem |  |  |
| 104 | VvCYP82C2m | VIT_212s0035g00610.1 | chr12:20009047..20011409 | reverse | 1560 | 519 | 129.51 | 4.98 | 1 | nucl:6,mito:5,cyto:2,extr:1 | Proximal |  |  |
| 105 | VvCYP82C2o | VIT_218s0001g11480.1 | chr18:9762292..9784846 | forward | 1482 | 493 | 122.39 | 4.99 | 2 | nucl:6,mito:5,cyto:2,extr:1 | Proximal |  |  |
| 106 | VvCYP82C4e | VIT_218s0001g11520.1 | chr18:9804770..9807880 | forward | 1578 | 525 | 130.66 | 4.98 | 2 | E.R.:5.5,E.R._plas:4,extr:3,cyto:2,mito:2,plas:1.5 | Proximal |  |  |
| 107 | VvCYP83B1 | VIT_209s0002g01090.1 | chr9:829306..832222 | forward | 1536 | 511 | 127.10 | 4.93 | 1 | plas:10,golg:2,cyto:1,vacu:1 | Dispersed | CYP83 |  |
| 108 | VvCYP84A1a | VIT_203s0038g00500.1 | chr3:456981..458989 | forward | 1554 | 517 | 126.92 | 4.98 | 1 | E.R.:5.5,E.R._plas:4,extr:3,cyto:2,mito:2,plas:1.5 | Proximal | CYP84 |  |
| 109 | VvCYP84A1b | VIT_203s0038g00550.1 | chr3:509995..512013 | forward | 1611 | 536 | 131.93 | 4.97 | 1 | E.R.:4.5,E.R._plas:3.5,cyto:3,extr:3,mito:2,plas:1.5 | Proximal |  |  |
| 110 | VvCYP84A1c | VIT_204s0023g02900.1 | chr4:19492533..19494780 | forward | 1545 | 514 | 127.46 | 4.96 | 1 | E.R.:4.5,cyto:3.5,E.R._plas:3.5,cyto_pero:2.5,mito:2,plas:1.5,chlo:1,extr:1 | Singleton |  |  |
| 111 | VvCYP89A5 | VIT_214s0036g00140.1 | chr14:9641932..9643200 | reverse | 1254 | 417 | 104.21 | 5.00 | 0 | E.R.:5.5,E.R._plas:4,extr:3,cyto:2,mito:2,plas:1.5 | Tandem | CYP89 |  |
| 112 | VvCYP89A2a | VIT_216s0039g00760.1 | chr16:401533..403436 | forward | 1620 | 539 | 133.44 | 4.95 | 0 | plas:9,golg:2,cyto:1,extr:1,vacu:1 | Proximal |  |  |
| 113 | VvCYP89A2b | VIT_216s0039g00820.1 | chr16:439597..441387 | forward | 1554 | 517 | 127.99 | 4.96 | 0 | plas:10,golg:2,cyto:1,vacu:1 | Proximal |  |  |
| 114 | VvCYP89A2c | VIT_216s0039g00840.1 | chr16:455280..458248 | forward | 1551 | 516 | 128.49 | 4.96 | 1 | E.R.:4.5,cyto:3.5,E.R._plas:3.5,cyto_pero:2.5,mito:2,plas:1.5,chlo:1,extr:1 | Proximal |  |  |
| 115 | VvCYP89A2d | VIT_216s0039g00860.1 | chr16:479387..481071 | forward | 1557 | 518 | 128.50 | 4.98 | 1 | plas:10,cyto:1,extr:1,vacu:1,golg:1 | Proximal |  |  |
| 116 | VvCYP89A2e | VIT_216s0039g00870.1 | chr16:484920..486817 | forward | 1674 | 557 | 138.05 | 4.96 | 1 | E.R.:5.5,E.R._plas:4,extr:3,cyto:2,mito:2,plas:1.5 | Proximal |  |  |
| 117 | VvCYP89A2f | VIT_216s0039g00880.1 | chr16:490051..491623 | reverse | 1551 | 516 | 128.47 | 4.99 | 1 | cyto:4.5,E.R.:3.5,cyto_pero:3,E.R._plas:3,mito:2,plas:1.5,chlo:1,extr:1 | Tandem |  |  |
| 118 | VvCYP89A2g | VIT_216s0039g00900.1 | chr16:494884..497275 | forward | 1581 | 526 | 129.60 | 4.97 | 0 | E.R.:4.5,cyto:3.5,E.R._plas:3.5,cyto_pero:2.5,mito:2,plas:1.5,chlo:1,extr:1 | Tandem |  |  |
| 119 | VvCYP89A2h | VIT_216s0039g00910.1 | chr16:497276..505083 | forward | 1566 | 521 | 128.44 | 4.97 | 0 | E.R.:4.5,cyto:3.5,E.R._plas:3.5,cyto_pero:2.5,mito:2,plas:1.5,chlo:1,extr:1 | Tandem |  |  |
| 120 | VvCYP89A2i | VIT_216s0039g00920.1 | chr16:506130..508048 | forward | 1566 | 521 | 129.20 | 4.97 | 1 | plas:8,vacu:3,cyto:1,extr:1,golg:1 | Tandem |  |  |
| 121 | VvCYP89A2j | VIT_216s0039g00930.1 | chr16:511725..513284 | forward | 1557 | 518 | 128.12 | 4.98 | 1 | plas:8,vacu:3,cyto:1,extr:1,golg:1 | Proximal |  |  |
| 122 | VvCYP89A2k | VIT_216s0039g01060.1 | chr16:575368..577060 | reverse | 1557 | 518 | 128.32 | 4.97 | 0 | plas:9,golg:2,cyto:1,extr:1,vacu:1 | Proximal |  |  |
| 123 | VvCYP89A2l | VIT_216s0039g01090.1 | chr16:595582..597540 | forward | 906 | 301 | 72.42 | 5.14 | 0 | nucl:5,mito:5,cyto:3,extr:1 | Proximal |  |  |
| 124 | VvCYP93D1a | VIT_208s0007g07710.1 | chr8:21171945..21174925 | reverse | 1581 | 526 | 130.69 | 4.99 | 1 | nucl:6,mito:5,cyto:2,extr:1 | Proximal | CYP93 |  |
| 125 | VvCYP93D1b | VIT_208s0007g07720.1 | chr8:21178877..21181204 | reverse | 1542 | 513 | 126.28 | 4.99 | 1 | E.R.:4.5,E.R._plas:4,extr:3,plas:2.5,cyto:2,mito:2 | Singleton |  |  |
| 126 | VvCYP93D1c | VIT_208s0007g07730.1 | chr8:21184277..21188606 | reverse | 1548 | 515 | 126.79 | 5.01 | 1 | plas:9,vacu:3,cyto:1,extr:1 | Tandem |  |  |
| 127 | VvCYP93D1d | VIT_208s0007g07740.1 | chr8:21190395..21194242 | reverse | 1551 | 516 | 127.12 | 5.01 | 1 | E.R.:5.5,E.R._plas:4,extr:3,cyto:2,mito:2,plas:1.5 | Tandem |  |  |
| 128 | VvCYP98A3 | VIT_208s0040g00780.1 | chr8:11733238..11736656 | reverse | 1527 | 508 | 125.78 | 4.97 | 0 | plas:7,vacu:3,extr:2,chlo:1,cyto:1 | Singleton | CYP98 |  |
| 129 | VvCYP706A3a | VIT_200s0555g00020.1 | chrUn:31688916..31691717 | reverse | 1593 | 530 | 129.66 | 4.97 | 1 | E.R.:5.5,E.R._plas:4,extr:3,cyto:2,mito:2,plas:1.5 | Dispersed | CYP706 | 79 |
| 130 | VvCYP706A4a | VIT_205s0094g01180.1 | chr5:24490035..24491947 | forward | 1113 | 370 | 89.49 | 5.10 | 0 | nucl:6,mito:5,cyto:2,extr:1 | Dispersed |  |  |
| 131 | VvCYP706A1 | VIT_219s0015g00070.1 | chr19:8035822..8037119 | reverse | 828 | 275 | 67.65 | 5.15 | 0 | nucl:6,mito:5,cyto:2,extr:1 | Proximal |  |  |
| 132 | VvCYP706A4b | VIT_205s0094g01190.1 | chr5:24497250..24500001 | forward | 1701 | 566 | 140.37 | 4.97 | 0 | plas:11,cyto:1,vacu:1,golg:1 | Tandem |  |  |
| 133 | VvCYP706A4d | VIT_216s0022g01390.1 | chr16:13378319..13382553 | forward | 1707 | 568 | 139.26 | 4.99 | 1 | nucl:5,mito:5,cyto:3,extr:1 | Singleton |  |  |
| 134 | VvCYP706A3b | VIT_216s0022g01500.1 | chr16:13531742..13534059 | reverse | 1605 | 534 | 132.38 | 4.99 | 1 | E.R.:4.5,E.R._plas:3.5,cyto:3,mito:2,extr:2,plas:1.5,chlo:1 | Tandem |  |  |
| 135 | VvCYP706A3c | VIT_216s0022g01540.1 | chr16:13618763..13620629 | reverse | 1119 | 372 | 91.22 | 5.08 | 0 | E.R.:5.5,E.R._plas:4,extr:3,cyto:2,mito:2,plas:1.5 | Proximal |  |  |
| 136 | VvCYP706A3d | VIT_216s0100g00550.1 | chr16:16038971..16040835 | forward | 1497 | 498 | 123.21 | 4.99 | 1 | plas:7,vacu:5,cyto:1,extr:1 | Tandem |  |  |
| 137 | VvCYP709B3 | VIT_207s0005g02490.1 | chr7:4846289..4849292 | reverse | 1578 | 525 | 129.50 | 5.00 | 1 | nucl:5,mito:5,cyto:3,extr:1 | Tandem | CYP709 | 72 |
| 138 | VvCYP714A1a | VIT_203s0167g00190.1 | chr3:12545432..12547480 | forward | 1533 | 510 | 125.41 | 4.99 | 1 | cyto:4.5,E.R.:3.5,cyto_pero:3,E.R._plas:3,mito:2,plas:1.5,chlo:1,extr:1 | Dispersed | CYP714 |  |
| 139 | VvCYP714A1b | VIT_213s0067g00110.1 | chr13:97734..102604 | forward | 1578 | 525 | 129.88 | 4.98 | 1 | nucl:5,mito:5,cyto:3,extr:1 | Tandem |  |  |
| 140 | VvCYP714A1c | VIT_218s0089g00700.1 | chr18:28531502..28533893 | forward | 1596 | 531 | 129.77 | 4.99 | 1 | plas:6,vacu:5,extr:2,cyto:1 | Tandem |  |  |
| 141 | VvCYP714A1d | VIT_215s0021g01060.1 | chr15:11091115..11096309 | forward | 1554 | 517 | 127.73 | 5.01 | 1 | nucl:7,mito:7 | Tandem |  |  |
| 142 | VvCYP715A1 | VIT_216s0050g00480.1 | chr16:17352540..17355775 | forward | 1554 | 517 | 128.42 | 4.99 | 2 | mito:6,nucl:5,cyto:2,chlo:1 | Singleton | CYP715 |  |
| 143 | VvCYP72A10a | VIT_200s0389g00030.1 | chrUn:27093936..27097365 | forward | 1542 | 513 | 126.63 | 5.01 | 1 | nucl:6,mito:5,cyto:2,extr:1 | Tandem | CYP72 |  |
| 144 | VvCYP72A11a | VIT_204s0008g01860.1 | chr4:1479623..1482644 | forward | 1539 | 512 | 126.33 | 5.00 | 1 | nucl:6,mito:5,cyto:2,extr:1 | Dispersed |  |  |
| 145 | VvCYP72A10b | VIT_211s0016g01290.1 | chr11:1036852..1039885 | reverse | 1062 | 353 | 87.61 | 5.08 | 0 | E.R.:5.5,E.R._plas:4,extr:3,cyto:2,mito:2,plas:1.5 | Tandem |  |  |
| 146 | VvCYP72A10c | VIT_219s0015g02050.1 | chr19:11236194..11240909 | forward | 1551 | 516 | 23.48 | 5.31 | 1 | E.R.:4.5,cyto:3.5,E.R._plas:3.5,cyto_pero:2.5,mito:2,plas:1.5,chlo:1,extr:1 | Dispersed |  |  |
| 147 | VvCYP72A10d | VIT_219s0015g02090.1 | chr19:11317331..11333144 | reverse | 1548 | 515 | 126.80 | 5.02 | 1 | E.R.:5.5,E.R._plas:4,extr:3,cyto:2,mito:2,plas:1.5 | Tandem |  |  |
| 148 | VvCYP72A10e | VIT_219s0015g02140.1 | chr19:11491570..11505169 | reverse | 1227 | 408 | 100.53 | 5.07 | 0 | E.R.:4.5,E.R._plas:3.5,cyto:3,mito:2,extr:2,plas:1.5,chlo:1 | Tandem |  |  |
| 149 | VvCYP72A10h | VIT_219s0015g02440.1 | chr19:12274448..12279547 | reverse | 1245 | 414 | 101.76 | 5.07 | 0 | E.R.:4.5,E.R._plas:3.5,cyto:3,mito:2,extr:2,plas:1.5,chlo:1 | Tandem |  |  |
| 150 | VvCYP72A10j | VIT_219s0015g02500.1 | chr19:12496267..12500336 | reverse | 1548 | 515 | 126.99 | 5.03 | 1 | E.R.:4.5,E.R._plas:3.5,cyto:3,mito:2,extr:2,plas:1.5,chlo:1 | WGD or segmental |  |  |
| 151 | VvCYP72A10k | VIT_219s0015g02520.1 | chr19:12608039..12612141 | forward | 1380 | 459 | 113.40 | 5.04 | 0 | nucl:6,mito:5,cyto:2,extr:1 | WGD or segmental |  |  |
| 152 | VvCYP72A11c | VIT_219s0015g02660.1 | chr19:12924172..12928072 | forward | 1371 | 456 | 112.76 | 5.04 | 0 | E.R.:5.5,E.R._plas:4,extr:3,cyto:2,mito:2,plas:1.5 | WGD or segmental |  |  |
| 153 | VvCYP72A1m | VIT_219s0015g02780.1 | chr19:13198267..13202394 | forward | 1548 | 515 | 127.09 | 5.02 | 1 | nucl:6,mito:5,cyto:2,extr:1 | WGD or segmental |  |  |
| 154 | VvCYP72A10n | VIT_219s0015g02900.1 | chr19:13551172..13555212 | forward | 1371 | 456 | 112.97 | 5.04 | 0 | vacu:7,plas:4,extr:2,cyto:1 | WGD or segmental |  |  |
| 155 | VvCYP72A10o | VIT_219s0015g02910.1 | chr19:13603170..13608539 | forward | 1545 | 514 | 126.90 | 5.03 | 1 | E.R.:4.5,E.R._plas:3.5,cyto:3,mito:2,extr:2,plas:1.5,chlo:1 | Tandem |  |  |
| 156 | VvCYP72A11d | VIT_219s0135g00120.1 | chr19:14894719..14897564 | forward | 1197 | 398 | 97.86 | 5.08 | 1 | nucl:6,mito:5,cyto:2,extr:1 | WGD or segmental |  |  |
| 157 | VvCYP72A10q | VIT_219s0135g00130.1 | chr19:14902297..14924143 | forward | 1557 | 518 | 128.06 | 5.02 | 1 | nucl:6,mito:5,cyto:2,extr:1 | WGD or segmental |  |  |
| 158 | VvCYP72A10r | VIT_219s0135g00150.1 | chr19:14954485..14958013 | forward | 1230 | 409 | 101.31 | 5.07 | 0 | nucl:6,mito:5,cyto:2,extr:1 | Tandem |  |  |
| 159 | VvCYP72A10s | VIT_219s0135g00190.1 | chr19:15027298..15032098 | reverse | 852 | 283 | 69.04 | 5.15 | 0 | plas:7,vacu:3,extr:2,chlo:1,cyto:1 | WGD or segmental |  |  |
| 160 | VvCYP72A10t | VIT_219s0135g00230.1 | chr19:15083914..15088984 | reverse | 1551 | 516 | 127.68 | 5.02 | 1 | E.R.:4.5,cyto:3.5,E.R._plas:3.5,cyto_pero:2.5,mito:2,plas:1.5,chlo:1,extr:1 | WGD or segmental |  |  |
| 161 | VvCYP72A10u | VIT_219s0135g00290.1 | chr19:15176651..15182380 | reverse | 1254 | 417 | 102.30 | 5.07 | 1 | E.R.:4.5,E.R._plas:3.5,cyto:3,extr:3,mito:2,plas:1.5 | WGD or segmental |  |  |
| 162 | VvCYP72A10v | VIT_200s0389g00040.1 | chrUn:27098469..27101921 | forward | 1542 | 513 | 126.22 | 5.02 | 1 | nucl:5,mito:5,cyto:3,extr:1 | Tandem |  |  |
| 163 | VvCYP72A10i | VIT_219s0015g02440.2 | chr19:12274448..12279547 | reverse | 864 | 287 | 69.99 | 5.15 | 0 | E.R.:4.5,E.R._plas:3.5,cyto:3,mito:2,extr:2,plas:1.5,chlo:1 | WGD or segmental |  |  |
| 164 | VvCYP721A1a | VIT_218s0001g12170.1 | chr18:10346183..10349031 | reverse | 825 | 274 | 66.97 | 5.13 | 1 | nucl:6,mito:5,cyto:2,extr:1 | Tandem | CYP721 |  |
| 165 | VvCYP721A1d | VIT_218s0001g12200.1 | chr18:10363344..10366975 | reverse | 1512 | 503 | 123.87 | 5.00 | 1 | E.R.:4.5,cyto:3.5,E.R._plas:3.5,cyto_pero:2.5,mito:2,plas:1.5,chlo:1,extr:1 | Tandem |  |  |
| 166 | VvCYP734A1a | VIT_209s0002g05980.1 | chr9:5785771..5789100 | forward | 1581 | 526 | 131.86 | 4.96 | 0 | nucl:7,mito:6,chlo:1 | Tandem | CYP734 |  |
| 167 | VvCYP734A1b | VIT_211s0016g04810.1 | chr11:4100613..4105250 | forward | 1596 | 531 | 132.21 | 4.96 | 1 | nucl:5,mito:5,cyto:3,extr:1 | Tandem |  |  |
| 168 | VvCYP735A1 | VIT_214s0006g02970.1 | chr14:21269249..21272044 | reverse | 1548 | 515 | 128.10 | 4.96 | 0 | E.R.:4.5,cyto:3.5,E.R._plas:3.5,cyto_pero:2.5,mito:2,plas:1.5,chlo:1,extr:1 | Tandem | CYP735 |  |
| 169 | VvCYP74A1a | VIT_203s0063g01820.1 | chr3:5156485..5158183 | reverse | 1464 | 487 | 122.93 | 4.98 | 0 | nucl:6,mito:5,cyto:2,extr:1 | Tandem | CYP74 | 74 |
| 170 | VvCYP74A1b | VIT_203s0063g01850.1 | chr3:5183292..5184849 | reverse | 1452 | 483 | 122.33 | 4.98 | 0 | E.R.:4.5,cyto:3.5,E.R._plas:3.5,cyto_pero:2.5,mito:2,plas:1.5,chlo:1,extr:1 | Tandem |  |  |
| 171 | VvCYP74A1c | VIT_203s0063g01860.1 | chr3:5192873..5194434 | reverse | 1452 | 483 | 122.19 | 4.98 | 0 | cyto:4.5,E.R.:3.5,cyto_pero:3,E.R._plas:3,mito:2,plas:1.5,chlo:1,extr:1 | Tandem |  |  |
| 172 | VvCYP74B2 | VIT_212s0059g01060.1 | chr12:5972189..5975957 | reverse | 1464 | 487 | 124.18 | 4.92 | 0 | plas:9,golg:2,cyto:1,extr:1,vacu:1 | Singleton |  |  |
| 173 | VvCYP74A1d | VIT_218s0001g11630.1 | chr18:9911196..9912972 | reverse | 1563 | 520 | 132.15 | 4.93 | 0 | plas:9,golg:2,cyto:1,extr:1,vacu:1 | Singleton |  |  |
| 174 | VvCYP74A1e | VIT_203s0063g01830.1 | chr3:5160926..5173965 | reverse | 1719 | 572 | 144.14 | 4.94 | 0 | plas:8,vacu:3,cyto:1,extr:1,golg:1 | Tandem |  |  |
| 175 | VvCYP702A2 | VIT_218s0001g00030.1 | chr18:1084705..1087799 | reverse | 1020 | 339 | 84.99 | 5.11 | 2 | nucl:7,mito:6,chlo:1 | Proximal | CYP702 | 85 |
| 176 | VvCYP707A1b | VIT_203s0063g00380.1 | chr3:3944372..3947589 | forward | 1518 | 505 | 125.94 | 5.00 | 1 | nucl:6,mito:5,cyto:2,extr:1 | Tandem | CYP707 |  |
| 177 | VvCYP707A1c | VIT_206s0004g05050.1 | chr6:5973017..5977723 | reverse | 1467 | 488 | 123.04 | 4.97 | 1 | plas:10,cyto:1,extr:1,vacu:1,golg:1 | Tandem |  |  |
| 178 | VvCYP707A1d | VIT_207s0031g00690.1 | chr7:16852540..16859877 | forward | 1485 | 494 | 122.68 | 5.01 | 1 | E.R.:4.5,cyto:3.5,E.R._plas:3.5,cyto_pero:2.5,mito:2,plas:1.5,chlo:1,extr:1 | Singleton |  |  |
| 179 | VvCYP707A1e | VIT_218s0001g10500.1 | chr18:8847753..8850989 | forward | 1413 | 470 | 116.79 | 5.00 | 1 | plas:10,cyto:1,extr:1,vacu:1,golg:1 | Tandem |  |  |
| 180 | VvCYP708A1a | VIT_215s0048g01960.1 | chr15:16085960..16088517 | forward | 897 | 298 | 73.66 | 5.14 | 0 | mito:6,nucl:5,cyto:2,chlo:1 | Tandem | CYP708 |  |
| 181 | VvCYP708A2 | VIT_216s0100g00600.1 | chr16:16088417..16091110 | forward | 1458 | 485 | 120.30 | 5.02 | 1 | nucl:7,mito:6,chlo:1 | Tandem |  |  |
| 182 | VvCYP716A1a | VIT_204s0008g02960.1 | chr4:2470144..2472805 | forward | 1449 | 482 | 120.53 | 4.97 | 1 | E.R.:4.5,E.R._plas:4,extr:3,plas:2.5,cyto:2,mito:2 | Tandem | CYP716 |  |
| 183 | VvCYP716A2a | VIT_211s0065g00040.1 | chr11:13104932..13108116 | forward | 1149 | 382 | 96.99 | 5.01 | 1 | E.R.:4.5,E.R._plas:3.5,cyto:3,extr:3,mito:2,plas:1.5 | Dispersed |  |  |
| 184 | VvCYP716A1b | VIT_211s0065g00130.1 | chr11:13427386..13430454 | forward | 1149 | 382 | 96.90 | 5.01 | 1 | E.R.:4.5,E.R._plas:3.5,cyto:3,extr:3,mito:2,plas:1.5 | Dispersed |  |  |
| 185 | VvCYP716A1c | VIT_211s0065g00630.1 | chr11:14524425..14527262 | forward | 1596 | 531 | 132.76 | 4.98 | 2 | E.R.:4.5,E.R._plas:4,cyto:3,plas:2.5,mito:2,extr:2 | Singleton |  |  |
| 186 | VvCYP716A1e | VIT_218s0041g01810.1 | chr18:26756577..26758590 | reverse | 1284 | 427 | 106.76 | 5.04 | 0 | nucl:6,mito:5,cyto:2,extr:1 | Tandem |  |  |
| 187 | VvCYP716A1g | VIT_218s0072g00580.1 | chr18:19797952..19801656 | reverse | 1443 | 480 | 121.56 | 4.96 | 1 | plas:8,vacu:3,cyto:1,extr:1,golg:1 | Dispersed |  |  |
| 188 | VvCYP716A1i | VIT_210s0092g00070.1 | chr10:11138600..11140198 | reverse | 1428 | 475 | 118.15 | 4.98 | 1 | plas:11,cyto:1,extr:1,vacu:1 | Singleton |  |  |
| 189 | VvCYP716A1j | VIT_215s0024g01830.1 | chr15:3854728..3856682 | reverse | 1158 | 385 | 96.78 | 5.05 | 1 | E.R.:4.5,E.R._plas:3.5,cyto:3,mito:2,extr:2,plas:1.5,chlo:1 | Dispersed |  |  |
| 190 | VvCYP716A2b | VIT_218s0041g01800.1 | chr18:26733761..26735477 | forward | 1452 | 483 | 120.67 | 4.99 | 1 | plas:9,vacu:3,cyto:1,extr:1 | Tandem |  |  |
| 191 | VvCYP720A1 | VIT_216s0039g00290.1 | chr16:140769..143178 | reverse | 1461 | 486 | 119.96 | 5.03 | 1 | mito:6,nucl:5,cyto:2,chlo:1 | Singleton | CYP720 |  |
| 192 | VvCYP722A1a | VIT_218s0001g12870.1 | chr18:10967830..10971646 | reverse | 1509 | 502 | 124.20 | 4.99 | 1 | nucl:6,mito:5,cyto:2,extr:1 | Tandem | CYP722 |  |
| 193 | VvCYP722A1b | VIT_204s0023g02350.1 | chr4:18887796..18891508 | reverse | 1389 | 462 | 113.53 | 5.04 | 0 | nucl:6,mito:5,cyto:2,extr:1 | Singleton |  |  |
| 194 | VvCYP724A1 | VIT_214s0066g00170.1 | chr14:26743582..26745706 | forward | 1197 | 398 | 98.39 | 5.05 | 0 | E.R.:4.5,E.R._plas:4,cyto:3,mito:3,plas:2.5,extr:1 | Singleton | CYP724 |  |
| 195 | VvCYP85A1a | VIT_201s0011g00170.1 | chr1:263078..269146 | reverse | 885 | 294 | 72.52 | 5.11 | 0 | nucl:6,mito:5,cyto:2,extr:1 | Tandem | CYP85 |  |
| 196 | VvCYP85A1b | VIT_201s0011g00190.1 | chr1:284363..287490 | reverse | 1392 | 463 | 114.13 | 5.03 | 1 | nucl:7,mito:7 | Tandem |  |  |
| 197 | VvCYP85A1c | VIT_214s0083g01110.1 | chr14:23435304..23438610 | reverse | 1152 | 383 | 94.81 | 5.05 | 0 | nucl:6,mito:5,cyto:2,extr:1 | Tandem |  |  |
| 198 | VvCYP87A2a | VIT_202s0025g04080.1 | chr2:3590917..3594823 | forward | 1140 | 379 | 93.61 | 5.08 | 0 | nucl:6,mito:5,cyto:2,extr:1 | Tandem | CYP87 |  |
| 199 | VvCYP87A2b | VIT_216s0100g00610.1 | chr16:16095996..16099561 | forward | 1440 | 479 | 118.66 | 5.02 | 1 | nucl:7,mito:6,chlo:1 | Singleton |  |  |
| 200 | VvCYP87A2c | VIT_218s0122g01460.1 | chr18:1007917..1011014 | forward | 1431 | 476 | 118.64 | 5.03 | 2 | nucl:7,mito:6,chlo:1 | Proximal |  |  |
| 201 | VvCYP87A2d | VIT_218s0122g01470.1 | chr18:1039528..1042444 | reverse | 1431 | 476 | 118.62 | 5.04 | 2 | nucl:7,mito:6,chlo:1 | Tandem |  |  |
| 202 | VvCYP87A2e | VIT_218s0122g01480.1 | chr18:1054582..1057679 | reverse | 1320 | 439 | 109.41 | 5.06 | 0 | nucl:6,mito:5,cyto:2,extr:1 | Tandem |  |  |
| 203 | VvCYP88A3a | VIT_215s0021g00270.1 | chr15:9762823..9768728 | reverse | 1479 | 492 | 121.56 | 5.02 | 0 | nucl:6,mito:6,chlo:1,cyto:1 | Tandem | CYP88 |  |
| 204 | VvCYP88A3b | VIT_215s0021g00310.1 | chr15:9799146..9801559 | reverse | 1467 | 488 | 120.21 | 5.01 | 1 | nucl:7,mito:6,chlo:1 | Tandem |  |  |
| 205 | VvCYP90B1a | VIT_204s0023g01630.1 | chr4:18166904..18171539 | forward | 1077 | 358 | 88.66 | 5.07 | 1 | nucl:5,mito:5,cyto:3,extr:1 | Tandem | CYP90 |  |
| 206 | VvCYP90C1a | VIT_204s0023g02650.1 | chr4:19189216..19194688 | forward | 1461 | 486 | 118.77 | 5.01 | 0 | cyto:5.5,cyto_pero:3.5,mito:2,E.R.:2,chlo:1,nucl:1,plas:1,extr:1 | Tandem |  |  |
| 207 | VvCYP90C1b | VIT_209s0002g02080.1 | chr9:1886480..1892105 | reverse | 1428 | 475 | 118.09 | 5.00 | 1 | E.R.:4.5,E.R._plas:3.5,cyto:3,mito:2,extr:2,plas:1.5,chlo:1 | Tandem |  |  |
| 208 | VvCYP90A1 | VIT_213s0067g00660.1 | chr13:359857..364350 | forward | 1458 | 485 | 120.55 | 4.96 | 1 | nucl:6,mito:5,cyto:2,extr:1 | Tandem |  |  |
| 209 | VvCYP90B1b | VIT_212s0057g01460.1 | chr12:10162753..10165832 | reverse | 1419 | 472 | 118.91 | 5.01 | 1 | cyto:4.5,E.R.:3.5,cyto_pero:3,E.R._plas:3,mito:2,plas:1.5,chlo:1,extr:1 | Singleton |  |  |
| 210 | VvCYP704A2 | VIT_201s0026g02700.1 | chr1:12438183..12440475 | reverse | 1440 | 479 | 119.30 | 5.01 | 0 | nucl:6,mito:5,cyto:2,extr:1 | Singleton | CYP704 | 86 |
| 211 | VvCYP704A1a | VIT_215s0048g01670.1 | chr15:15868804..15872017 | reverse | 1548 | 515 | 127.28 | 5.01 | 1 | nucl:6,mito:5,cyto:2,extr:1 | Tandem |  |  |
| 212 | VvCYP704A1b | VIT_215s0048g01680.1 | chr15:15882456..15885257 | reverse | 1548 | 515 | 127.25 | 5.01 | 1 | nucl:6,mito:5,cyto:2,extr:1 | Tandem |  |  |
| 213 | VvCYP704A1c | VIT_215s0048g01690.1 | chr15:15892125..15895019 | reverse | 1587 | 528 | 130.71 | 5.00 | 1 | nucl:5,mito:5,cyto:3,extr:1 | Tandem |  |  |
| 214 | VvCYP704A1d | VIT_215s0048g01700.1 | chr15:15906545..15909573 | reverse | 1554 | 517 | 129.55 | 4.98 | 1 | plas:8,vacu:3,cyto:1,extr:1,golg:1 | Tandem |  |  |
| 215 | VvCYP704A1e | VIT_216s0022g02140.1 | chr16:14575868..14580202 | forward | 1461 | 486 | 120.20 | 5.06 | 0 | mito:6,nucl:5,cyto:2,chlo:1 | Tandem |  |  |
| 216 | VvCYP704A1f | VIT_216s0022g02150.1 | chr16:14592615..14594689 | forward | 1503 | 500 | 123.74 | 5.01 | 2 | nucl:5,mito:5,cyto:3,extr:1 | Singleton |  |  |
| 217 | VvCYP86B1a | VIT_201s0011g02060.1 | chr1:1748090..1751655 | forward | 1638 | 545 | 134.23 | 4.97 | 1 | nucl:6,mito:5,cyto:2,extr:1 | Singleton | CYP86 |  |
| 218 | VvCYP86C1 | VIT_201s0137g00410.1 | chr1:6936328..6938135 | forward | 1683 | 560 | 137.87 | 4.99 | 3 | E.R.:3.5,cyto:3,mito:3,E.R._plas:3,plas:1.5,chlo:1,nucl:1,extr:1 | Dispersed |  |  |
| 219 | VvCYP86A1a | VIT_202s0025g03320.1 | chr2:2829143..2833459 | forward | 1629 | 542 | 134.91 | 4.94 | 1 | E.R.:4.5,E.R._plas:4,cyto:3,plas:2.5,mito:2,extr:2 | Dispersed |  |  |
| 220 | VvCYP86B1b | VIT_203s0017g01070.1 | chr3:16640517..16642082 | reverse | 1497 | 498 | 123.13 | 5.01 | 1 | mito:6,nucl:5,cyto:2,chlo:1 | Dispersed |  |  |
| 221 | VvCYP86A1b | VIT_206s0004g06210.1 | chr6:7005633..7007477 | forward | 1560 | 519 | 129.44 | 4.94 | 1 | E.R.:5.5,E.R._plas:4,extr:3,cyto:2,mito:2,plas:1.5 | Dispersed |  |  |
| 222 | VvCYP86A1c | VIT_215s0046g02380.1 | chr15:19173905..19175788 | forward | 1701 | 566 | 139.76 | 4.94 | 1 | nucl:7,mito:6,chlo:1 | Dispersed |  |  |
| 223 | VvCYP94C1a | VIT_206s0080g00090.1 | chr6:19789442..19791139 | reverse | 1338 | 445 | 110.07 | 5.00 | 0 | nucl:6,mito:5,cyto:2,extr:1 | Singleton | CYP94 |  |
| 224 | VvCYP94C1b | VIT_208s0007g03660.1 | chr8:17604366..17606629 | reverse | 1650 | 549 | 138.11 | 4.96 | 1 | cyto:4.5,E.R.:3.5,cyto_pero:3,E.R._plas:3,mito:2,plas:1.5,chlo:1,extr:1 | Singleton |  |  |
| 225 | VvCYP94D1 | VIT_208s0040g00820.1 | chr8:11777899..11779610 | forward | 1503 | 500 | 124.10 | 4.97 | 0 | plas:9,vacu:3,cyto:1,extr:1 | Singleton |  |  |
| 226 | VvCYP94C1c | VIT_213s0074g00790.1 | chr13:9237993..9239486 | reverse | 1410 | 469 | 117.25 | 5.00 | 0 | E.R.:4.5,cyto:3.5,E.R._plas:3.5,cyto_pero:2.5,mito:2,plas:1.5,chlo:1,extr:1 | Singleton |  |  |
| 227 | VvCYP94B1a | VIT_214s0068g02120.1 | chr14:25721500..25723129 | forward | 1611 | 536 | 132.22 | 4.95 | 1 | plas:8,vacu:3,cyto:1,extr:1,golg:1 | Singleton |  |  |
| 228 | VvCYP94B1b | VIT_217s0000g01490.1 | chr17:1060849..1062539 | forward | 1539 | 512 | 125.41 | 4.97 | 1 | nucl:7,mito:6,chlo:1 | Singleton |  |  |
| 229 | VvCYP96A1a | VIT_207s0031g01660.1 | chr7:17757615..17760864 | reverse | 1509 | 502 | 124.24 | 5.01 | 0 | nucl:6,mito:5,cyto:2,extr:1 | Tandem | CYP96 |  |
| 230 | VvCYP96A1b | VIT_207s0031g01670.1 | chr7:17764334..17769272 | reverse | 1500 | 499 | 122.93 | 5.00 | 1 | nucl:6,mito:5,cyto:2,extr:1 | Tandem |  |  |
| 231 | VvCYP96A1c | VIT_207s0031g01680.1 | chr7:17778943..17780654 | reverse | 1524 | 507 | 124.85 | 4.99 | 2 | nucl:6,mito:5,cyto:2,extr:1 | Tandem |  |  |
| 232 | VvCYP96A1d | VIT_214s0006g01670.1 | chr14:18141196..18142477 | forward | 1275 | 424 | 105.96 | 5.03 | 0 | nucl:6,mito:5,cyto:2,extr:1 | Tandem |  |  |
| 233 | VvCYP97B3 | VIT_203s0017g01930.1 | chr3:18343089..18371691 | reverse | 1719 | 572 | 142.13 | 4.99 | 0 | nucl:6,mito:6,chlo:1,cyto:1 | Tandem | CYP97 |  |
| 234 | VvCYP97C1 | VIT_208s0007g04530.1 | chr8:18449431..18457877 | reverse | 1641 | 546 | 137.27 | 4.98 | 0 | plas:10,chlo:1,cyto:1,extr:1,vacu:1 | Singleton |  |  |
| 235 | VvCYP710A1 | VIT_210s0003g03170.1 | chr10:5306982..5309170 | reverse | 1530 | 509 | 128.33 | 4.93 | 1 | mito:6,nucl:5,cyto:2,chlo:1 | Singleton | CYP710 | 710 |
| 236 | VvCYP711A1b | VIT_204s0008g01100.1 | chr4:916045..919492 | reverse | 1605 | 534 | 134.18 | 4.96 | 1 | E.R.:5.5,E.R._plas:4,extr:3,cyto:2,mito:2,plas:1.5 | Singleton | CYP711 | 711 |

^a^Systematic designation given to grapevine CYP genes

^b^The theoretical isoelectric points(pI)and molecular weights(MW)of the deduced polypeptides were calculated using the ExPASy Compute pI/Mw tool(<http://expasy.org/>)

^c^The number of transmembrane domains was predicted by TMHMMServerv2.0.

^d^The subcellular localizations were predicted by WoLFPSORT. plas, plasmamembrane; vacu, vacuolarmembrane; chlo, chloroplast, nucl, nucleus; E.R., endoplasmatic reticulum; cyto, cytosol; golg, Golgi.
