# Supplementary figures and images for: The Cytochrome P450 Monooxygenase Inventory of Grapevine (Vitis vinifera L.): Genome-Wide Identification, Evolutionary Characterization and Expression Analysis
Source: Front Genet. 2020 Feb 18;11:44. doi: 10.3389/fgene.2020.00044 (PMC7040366; doi:10.3389/fgene.2020.00044)

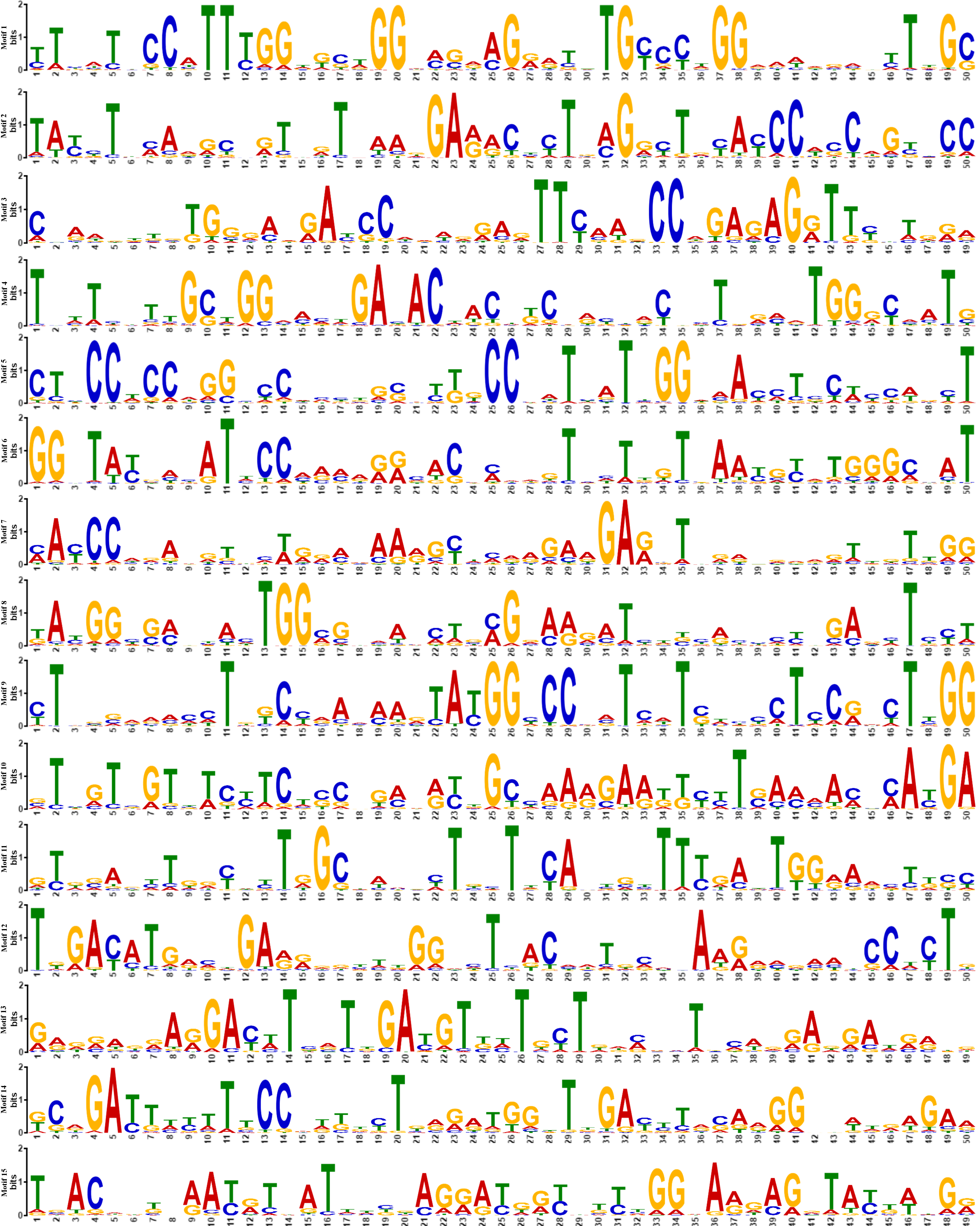

Supplement: Supplementary file 5 [file Image_1.png]

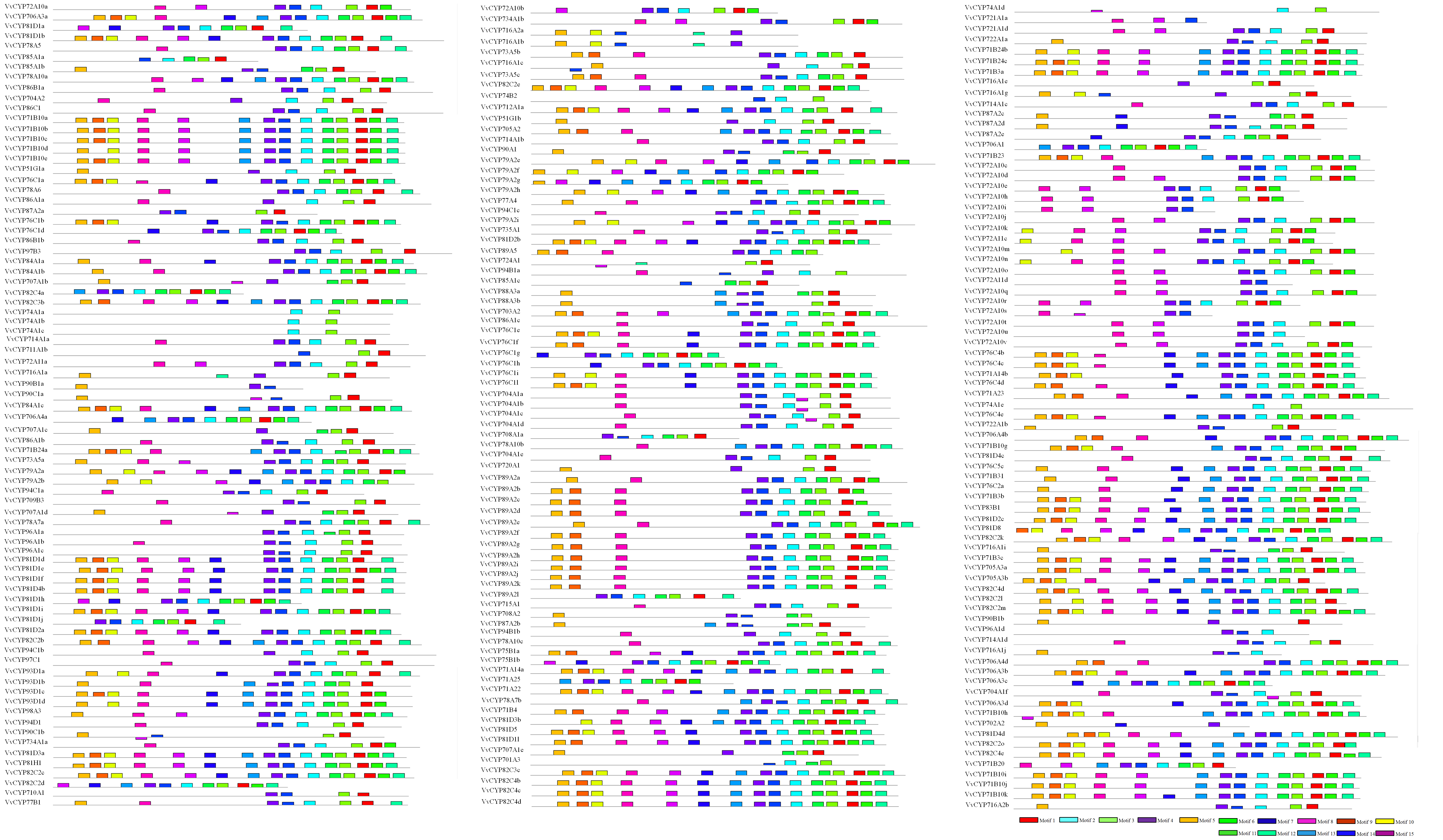

Supplement: Supplementary file 6 [file Image_2.png]

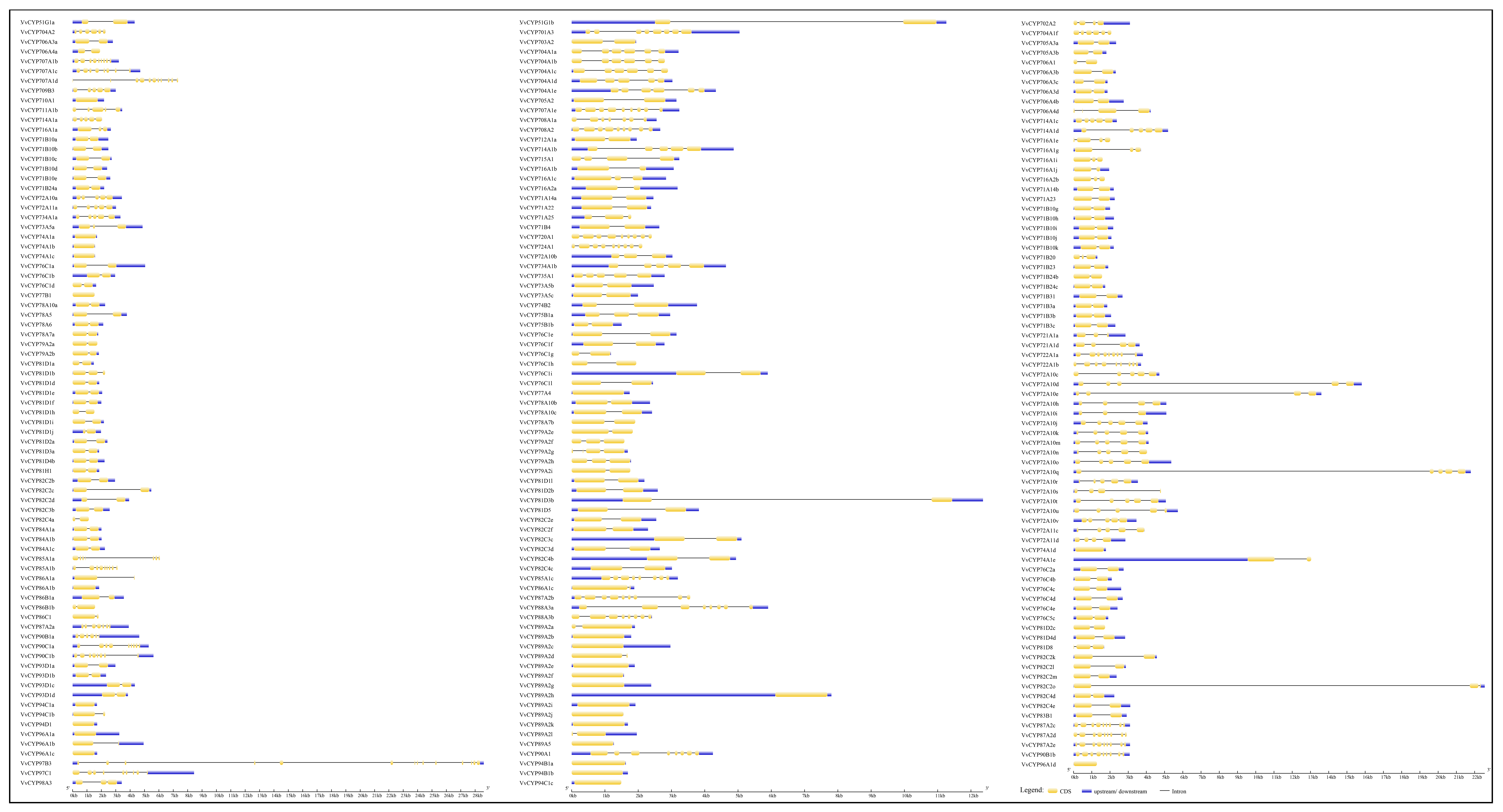

Supplement: Supplementary file 7 [file Image_3.tif]
